# Single-cell RNA sequencing data imputation using bi-level feature propagation

**DOI:** 10.1093/bib/bbae209

**Published:** 2024-05-05

**Authors:** Junseok Lee, Sukwon Yun, Yeongmin Kim, Tianlong Chen, Manolis Kellis, Chanyoung Park

**Affiliations:** Department of Industrial and Systems Engineering, KAIST, 291 Daehak-ro, Yuseong-gu, Daejeon 34141, Republic of Korea; Department of Computer Science, 201 S. Columbia St. CB 3175, UNC-Chapel Hill, Chapel Hill, NC 27599, United States; School of Computing, KAIST, 291 Daehak-ro, Yuseong-gu, Daejeon 34141, Republic of Korea; Department of Computer Science, 201 S. Columbia St. CB 3175, UNC-Chapel Hill, Chapel Hill, NC 27599, United States; Computer Science and Artificial Intelligence Laboratory, Massachusetts Institute of Technology, 32 Vassar St, Cambridge, MA 02139, United States; Broad Institute of MIT and Harvard, Merkin Building, 415 Main St., Cambridge, MA 02142, United States; Computer Science and Artificial Intelligence Laboratory, Massachusetts Institute of Technology, 32 Vassar St, Cambridge, MA 02139, United States; Broad Institute of MIT and Harvard, Merkin Building, 415 Main St., Cambridge, MA 02142, United States; Department of Industrial and Systems Engineering, KAIST, 291 Daehak-ro, Yuseong-gu, Daejeon 34141, Republic of Korea

**Keywords:** scRNA-seq, imputation, feature propagation

## Abstract

Single-cell RNA sequencing (scRNA-seq) enables the exploration of cellular heterogeneity by analyzing gene expression profiles in complex tissues. However, scRNA-seq data often suffer from technical noise, dropout events and sparsity, hindering downstream analyses. Although existing works attempt to mitigate these issues by utilizing graph structures for data denoising, they involve the risk of propagating noise and fall short of fully leveraging the inherent data relationships, relying mainly on one of cell–cell or gene–gene associations and graphs constructed by initial noisy data. To this end, this study presents single-cell bilevel feature propagation (scBFP), two-step graph-based feature propagation method. It initially imputes zero values using non-zero values, ensuring that the imputation process does not affect the non-zero values due to dropout. Subsequently, it denoises the entire dataset by leveraging gene–gene and cell–cell relationships in the respective steps. Extensive experimental results on scRNA-seq data demonstrate the effectiveness of scBFP in various downstream tasks, uncovering valuable biological insights.

## INTRODUCTION

Single-cell RNA sequencing (scRNA-seq) has ushered in a transformative approach to gene expression analysis, enabling unprecedented resolution at the single-cell level. This technology has paved the way for groundbreaking discoveries, from the identification of novel cell types [1, 2] and the detection of marker genes [3] to the intricate analysis of cellular trajectories [4]. Yet, the analysis of scRNA-seq data can pose significant challenges owing to the inherent noise in the observed values caused by various factors like amplification bias and cell cycle effects [5]. Furthermore, due to the low RNA capture rate, unobserved values (i.e. zero values) encompass both true biological absence and technical omissions, the latter often termed as dropout. Such dropout phenomena compromise the performance of downstream analyses, particularly in cell clustering, thereby skewing biological interpretations.

In addressing the above challenges, contemporary imputation techniques for scRNA-seq data can be broadly categorized into ‘non-graph-based methods’ and ‘graph-based methods’. Within the realm of non-graph-based methods, notable contributions include SAVER [6], scImpute [7] and DCA [8]. These methods predominantly impute zeroes by drawing assistance from other genes or cells through either the statistical strategies [6, 7] or the autoencoder framework [8]. Nevertheless, these techniques often do not fully harness the underlying relationships among genes or cells, thereby missing out on potentially valuable insights from their proximate counterparts.

Building upon this foundation, graph-based techniques have been favored due to their proficiency in encapsulating the utilization of relationships. Prototypical methods in this category encompass MAGIC [9], scGNN [10] and scGCL [11]. They employ a graph-diffusion or message-passing scheme upon a cell graph, rooted in the premise that adjacent cells in the graph share similar underlying biological characteristics. However, a notable limitation remains, which involves the risk of propagating noise when diffusing zero values that might potentially be attributed to dropout phenomena. As this unintended noise propagation can blur biologically meaningful values, a meticulous imputation process must be carried out prior to the diffusion of zero values.

Moreover, current graph-based methods do not fully harness the relationship information embedded within scRNA-seq data. One noteworthy limitation is that these methods prioritize either cell–cell or gene–gene relationships, often neglecting the significance of the other. For instance, MAGIC, scGNN and scGCL emphasize intercellular relationships through their utilization of cell–cell graphs, and GraphSCI [12] concentrates on gene–gene relationships, missing opportunities to harness the relationships of genes and cells, respectively. A further limitation is that these methods solely rely on graph structures derived from initial sparse and potentially noisy raw data, neglecting enhancements during the imputation phase. We argue that this reliance on initial graph structure leads to sub-optimal results, as leveraging the imputed matrix can lead to enhanced graph structure, and conversely, an enhanced graph structure can contribute to better imputation outcomes.

**Figure 1:**
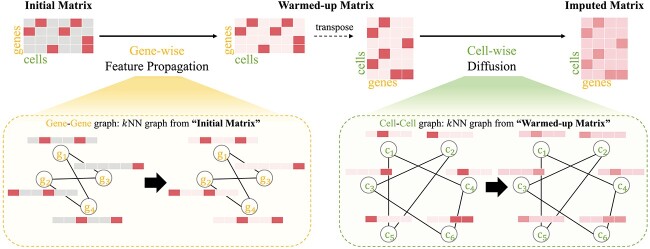
The overall framework of scBFP. Given an initial gene–cell count matrix, scBFP first conducts Gene-wise Feature Propagation on a gene–gene graph derived from this matrix, yielding a warmed-up matrix. Subsequently, using this warmed-up matrix, we derive an enhanced cell–cell graph and carry out diffusion to obtain the final imputed matrix.

Driven by these motivations, we introduce the single-cell bi-level feature propagation (scBFP). This is a novel framework that sequentially imputes zero values and denoises the entire counts in scRNA-seq datasets through an enhanced graph structure. Specifically, as depicted in Figure 1, scBFP first crafts a gene–gene interaction graph rooted in the raw count matrix and applies Feature Propagation [13, 14] to generate a ‘warmed-up’ matrix leveraging the gene–gene relationships. It is worth noting that scBFP addresses the dropout phenomena while maintaining non-zero values as their initial state during each iteration with simple replacement operation after propagation, thus preventing the possibility of noise diffusion from dropout events. Following this, scBFP constructs a graph in a cell perspective based on this ‘warmed-up’ matrix to obtain the enhanced graph structure. Finally, the imputed matrix is obtained by denoising the entire matrix, leveraging the diffusion process on this cell–cell graph. Through extensive experiments and comparisons with existing state-of-the-art imputation tools, we demonstrate that scBFP consistently delivers superior performance on various downstream tasks.

## METHODS

### The procedure of scBFP

In the quest to sequentially impute and denoise the cell–gene count matrix, scBFP employs feature propagation (FP) [13], a technique that preserves observed values (i.e. non-zeros) while imputing missing ones (i.e. zeros) through neighboring influences over iterative steps. Commencing with the initial gene–cell matrix $X \in \mathbb{R}^{G\times C}$, where $G$ and $C$ represent the total number of genes and cells, respectively, and a cosine-similarity-based k-nearest-neighbor gene–gene graph $\mathcal{G}^{\text{gene}}$, the aim of FP is to minimize the Dirichlet Energy across the cell expression of genes: $\ell (X,\mathcal{G}^{\text{gene}})= \sum _{c=1}^{C} \frac{1}{2}X_{\cdot ,c}^{\top }\Delta X_{\cdot ,c}$, with $\Delta \in \mathbb{R}^{G \times G} = I - \tilde{A}^{\text{gene}} (\tilde{A}^{\text{gene}} = D^{-1/2}A^{\text{gene}}D^{-1/2})$ representing the graph Laplacian matrix comprising a symmetrically normalized adjacency matrix $\tilde{A}^{\text{gene}}$ and a degree matrix $D$ with self-loops. This optimization essentially seeks to minimize differences between the features of interconnected genes.

The derivative of $X$ for the heat diffusion equation at time step $t$ is thus defined as: $\dot{X}(t)=-\nabla \ell (X(t))=-\Delta X(t)$, governed by the initial condition (IC): $X(0)=\left [ X_{\mathcal{N}}, X_{\mathcal{Z}}(0)\right ]^{\top }$ and the boundary condition (BC): $X_{\mathcal{N}}(t)=X_{\mathcal{N}}$, where subscripts $\mathcal{N}$ and $\mathcal{Z}$ designate the indices sets for non-zeros and zeros, respectively. The solution to this linear heat equation yields a closed-form solution, $X_{z}=-{\Delta }_{zz}^{-1}{\Delta }_{nz}^{\top }X_{n}$, which induces a complexity of $\mathcal{O}(|V_{z}|^{3})$, a cubic complexity of number of zero rows for solving linear equations—impractical for large graphs. Therefore, we resort to the iterative Euler scheme as follows: 


(1) \begin{align*}& \begin{aligned} {X}^{(i+1)} &= X^{(i)} - h \left[\begin{array}{cc} {0} & {0} \\{\Delta}_{zn} & {\Delta}_{zz} \end{array}\right]{X}^{(i)} \\ &= \left[\begin{array}{cc} {I} & {0} \\ -h{\Delta}_{zn} & I-h{\Delta}_{zz} \end{array}\right]{X}^{(i)} \end{aligned}\end{align*}

where $X^{(i)}$ represents the imputed gene-cell matrix at the $i$-th iteration, and $h$ signifies the step size within an iterative numerical scheme. Specifically, when $h=1$ and the graph Laplacian matrix is denoted by a normalized adjacency matrix, the iteration formula simplifies as follows: 


(2) \begin{align*}& {X}^{(i+1)} = \left[\begin{array}{cc} {I} & {0} \\ \tilde{{A}}_{zn}^{\text{gene}} & \tilde{{A}}_{zz}^{\text{gene}} \end{array}\right]{X}^{(i)}\end{align*}

With this equation, implementation is straightforward, involving the multiplication of the normalized adjacency matrix followed by the replacement of non-zero indices. After $I$ iterations, we acquire a ‘warmed-up’ gene–cell matrix $X^{(I)}$ in which the initially zero indices have been imputed based on gene–gene relationships. This matrix, regarded as an enhanced resource for cell-wise graph structure, is then transposed to shift focus to the cellular perspective, yielding a cell–gene matrix, $X^{\prime} \in \mathbb{R}^{C \times G} ={X^{(I)}}^{\top }$. Subsequently, we generate a cosine-similarity-based k-nearest-neighbor cell–cell graph, $\mathcal{G}^{\text{cell}}$, and conduct the final diffusion step as follows: 


(3) \begin{align*}& {X^{\prime}}^{(j+1)} = \tilde{{A}}^{\text{cell}} {X^{\prime}}^{(j)}\end{align*}

where ${X^{\prime}}^{(j)}$ represents the denoised cell-gene matrix at iteration $j$, and $\tilde{{A}}^{\text{cell}} \in \mathbb{R}^{C \times C} = D^{-1}A^{\text{cell}}$ is the random-walk based normalized (Here, compared to the gene–gene adjacency matrix, we employ a random-walk based normalization as spreading information from a high degree node to its neighbors is crucial. This is particularly significant for cells with sparsely captured gene expression, which can be enriched through the information from their high-degree neighboring cells.) cell–cell adjacency matrix. This cell-wise diffusion process not only imputes zero values in the initial matrix but also denoises non-zero values through neighboring cells, thereby fostering smoothness among similar cells. Following $J$ iterations, we ultimately obtain $\hat{X}\in \mathbb{R}^{C \times G}={X^{\prime}}^{(J)}$ as the final denoised matrix, poised for use in subsequent downstream tasks. The algorithm for scBFP can be found in Algorithm 1.


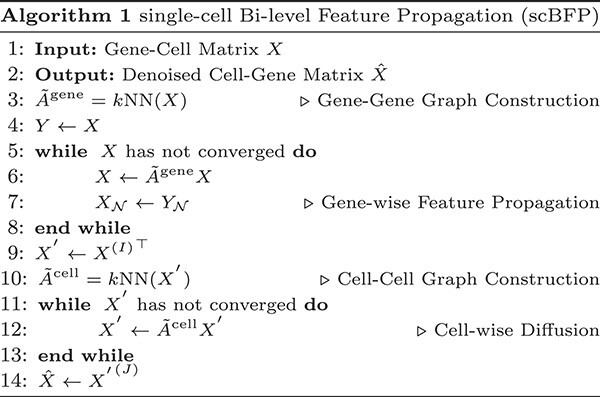


### The convergence property of scBFP

In our approach, we leverage the convergence property of imputed values facilitated by feature propagation. This aspect is crucial as it provides deeper insights into the variation and stabilization of imputed values across iterations, as delineated in Equation 2. We examine the convergence property from two distinct perspectives: the output of gene-wise feature propagation and cell-wise diffusion. These perspectives are integral to the sequential procedure of scBFP, aligning closely with its overall methodology. Given a gene-cell matrix, we begin with gene-wise feature propagation.

#### Convergence of gene-wise feature propagation

Proposition 1. (The output of Gene-wise Feature Propagation converges.) Take a symmetrically normalized adjacency matrix, i.e. $D^{-1/2}A^{gene}D^{-1/2}$, a strongly connected gene–gene graph $\tilde{A}^{gene}\in \mathbb{R}^{G\times G}$ with the gene-cell matrix $X \in \mathbb{R}^{G \times C}$, and $x \in \mathbb{R}^{G}$ be the gene vector. With $n$, $z$ being the index of nodes with non-zero values and zero values ($|n|+|z|=G$), respectively, define recursive iteration while $i$ ranging $\left [1,\infty \right )$ as

\begin{align*}& x^{(i)}=\left[\begin{array}{cc} I & \mathbf{0} \\ \tilde{A}^{gene}_{zn} & \tilde{A}^{gene}_{zz} \\ \end{array}\right]x^{(i-1)} \end{align*}

Then, this iteration converges with a steady state,

\begin{align*}& \lim_{i \rightarrow \infty} x^{(i)} =\left[\begin{array}{c} x_{n} \\ x_{z} \\ \end{array}\right] = \left[\begin{array}{c} x_{n} \\ -\Delta^{-1}_{zz}\tilde{A}^{gene}_{zn}x_{n} \end{array}\right] \end{align*}

Proof.
The iterative Euler scheme, which propagates features while maintaining initial states for the non-zero values in the gene-cell matrix, can be expressed as

\begin{align*}\begin{aligned} \left[\begin{array}{l} {x^{(i)}_{n}} \\{x_{z}^{(i)}} \\ \end{array}\right] &=\left[\begin{array}{cc} I_{|n|} & 0_{nz} \\ \tilde{A}_{zn}^{gene} & \tilde{A}^{gene}_{zz} \\ \end{array}\right]\left[\begin{array}{l} {x_{n}^{(i-1)}} \\{x_{z}^{(i-1)}} \\ \end{array}\right] \\ &=\left[\begin{array}{c} {x_{n}^{(i-1)}} \\ \tilde{A}_{nz}^{gene} {x_{n}^{(i-1)}}+\tilde{A}^{gene}_{zz} {\mathbf{x}_{z}^{(i-1)}}\\ \end{array}\right] \end{aligned} \end{align*}

Here, the first $|n|$ rows maintain their initial states from identity matrix $I$, $x_{n}^{(i)}= x_{n}^{(i-1)}=x_{n}$. This leaves only the rows with initially zero values,

\begin{align*}& {x_{z}^{(i)}} = \tilde{A}_{zn}^{gene} {x}_{n} + \tilde{A}_{zz}^{gene} {x_{z}^{(i-1)}} \end{align*}

By unrolling this recursion and taking the limit to evaluate the steady state,

\begin{align*}& \lim_{i \rightarrow \infty} {x_{z}^{(i)}} = \lim_{i \rightarrow \infty} (\tilde{A}_{zz}^{gene})^{i} {x_{z}^{(0)}} + (\sum_{j=1}^{i} \tilde{A}_{zz}^{{gene}^{(j-1)}}) \tilde{A}_{zn}^{gene} {x_{n}} \end{align*}

In this context, when considering $\tilde{A}_{zz}^{gene}$ as the bottom right submatrix of $\tilde{A}^{gene}$, its spectral radius becomes less than 1, in accordance with Lemma A.1 of [13], which results $\lim _{i \rightarrow \infty } (\tilde{A}_{zz}^{gene})^{i} {x_{z}^{(0)}}$ approaches 0. Also, since its eigenvalue is not 1, the eigenvalue of $I_{z} -\tilde{A}_{zz}^{gene}$ becomes non-zero, which becomes invertible. Finally, using the geometric series, the converged value of nodes with initially zero values in the gene-cell matrix can be obtained as

\begin{align*}& \lim_{i \rightarrow \infty} {x_{z}^{(i)}} =\left(I_{z}-\tilde{A}_{zz}^{gene}\right)^{-1} \tilde{A}_{zn}^{gene} {x_{n}} = -\Delta^{-1}_{zz}\tilde{A}^{gene}_{zn}x_{n} \end{align*}

A critical insight from **Proposition 1** is the mechanism through which the steady state of imputed values (i.e. $x_{z}$) is achieved. This state is reached predominantly through the influence of non-zero values (i.e. $x_{n}$), while the non-zero values themselves retain their initial state, unaffected by the zero values and thus preserving their original scale. Given that edges of genes are formed based on the similarity of their neighboring representations, it follows logically that the imputation of zero gene values occurs through their closely related neighbor genes. This underscores the intuitive and methodologically sound basis of our approach. Next, we show the convergence property of cell-wise diffusion.

#### Convergence of cell-wise diffusion

Proposition 2. (Likely Convergence of Cell-wise Diffusion output.) Take a random-walk-based normalized adjacency matrix, i.e. $D^{-1}A^{cell}$, a strongly connected cell–cell graph $\tilde{A}^{cell}\in \mathbb{R}^{C\times C}$ with the cell-gene matrix $X^{\prime} \in \mathbb{R}^{C \times G}$, and $x^{\prime} \in \mathbb{R}^{C}$ be the cell vector. Define recursive iteration while $j$ ranging $\left [1,\infty \right )$ as

\begin{align*}& x^{(j)}=\tilde{A}^{cell}x^{(j-1)} \end{align*}

Then, under typical conditions, this iteration is likely to converge to a stationary distribution $\pi $, satisfying

\begin{align*}& \pi = \tilde{A}^{cell} \pi \end{align*}

Proof.
Given that the cell–cell graph is strongly connected, it inherently possesses the property of irreducibility. This implies that it is possible to traverse from any node to any other node within the graph. Additionally, the graph exhibits ergodic characteristics, suggesting that the system represented by the matrix will not become confined to a subset of states but can eventually reach any state.

The convergence to a stationary distribution is generally expected when the largest eigenvalue of the transition matrix $\tilde{A}^{cell}$ is 1, and all other eigenvalues are ¡1 in absolute value. In many cases, especially in strongly connected graphs, the largest eigenvalue is typically 1, which supports the likelihood of convergence. This convergence property can often be empirically observed through stable performance across iterations.

In this study, we empirically observed that strong connectivity can be achieved with a sufficiently large value of $k$, typically exceeding 10. In summary, by understanding the convergence properties of both gene-wise feature propagation and cell-wise diffusion, we can leverage these characteristics to enhance the interpretability of the imputation process mechanism.

### Reproducibility of scBFP

In this study, the parameters of scBFP are set to the default parameter settings for all experiments, considering the unsupervised nature of the single-cell analysis. Detailed information regarding parameter and device configurations is provided in Supplementary Notes 1.1. For fair comparisons, the parameters of the baseline software are also set to those recommended in the official codes, the links to which are reported in Supplementary Table S2.

## RESULTS

### scBFP helps to conduct improved cell clustering

**Figure 2:**
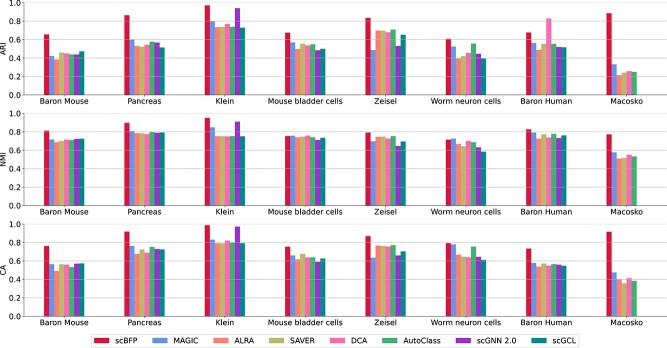
Performance comparisons of scBFP and other baselines on the eight scRNA-seq datasets.

To evaluate whether data imputed by scBFP can improve cell clustering performance, we conduct experiments with eight widely-used scRNA-seq datasets [15–21], all of which have gold-standard cell type information in Figure 2. Details for datasets and baselines are available in Supplementary Table S1 and S2, respectively.

Through these experiments, we have the following observations: (1) scBFP generally shows improved clustering performance compared to other baseline methods. To statistically verify the superior performance of scBFP, we conduct a one-sided Wilcoxon ranked-sum test across all datasets and provide the results in Supplementary Table S3. The results indicate that scBFP achieves significantly better clustering performance in terms of ARI, NMI and CA at the 95% confidence level. (2) Regardless of the complexity of deep-learning-based models, such as DCA, Autoclass, scGNN and scGCL, they do not generally outperform the other baselines. This highlights that increased model complexity does not always guarantee improved performance in the scRNA-seq domain. (3) On the other hand, MAGIC, which employs a graph-based diffusion strategy to harness cell–cell relationships, shows relatively strong performance. This demonstrates that leveraging relationship information is beneficial in the scRNA-seq analysis. However, it shows lower performance compared to scBFP because of the limitation that it imputes both zero and non-zero values simultaneously without considering the influence of dropout phenomena and does not utilize gene–gene relationships. (4) Graph-based deep-learning baselines (i.e. scGNN and scGCL) are not scalable to the Macosko dataset with 44 808 cells. It is due to the memory constraints caused by the fact that graph-based methods have to load all cell nodes and corresponding features (i.e. genes) on GPUs. It is worth noting that scBFP address this issue by proposing feature-wise batch propagation, leveraging the independence between features during the propagation step. By doing so, scBFP effectively addresses the memory issue while still harnessing the accelerated computational speed of GPU.

**Figure 3:**
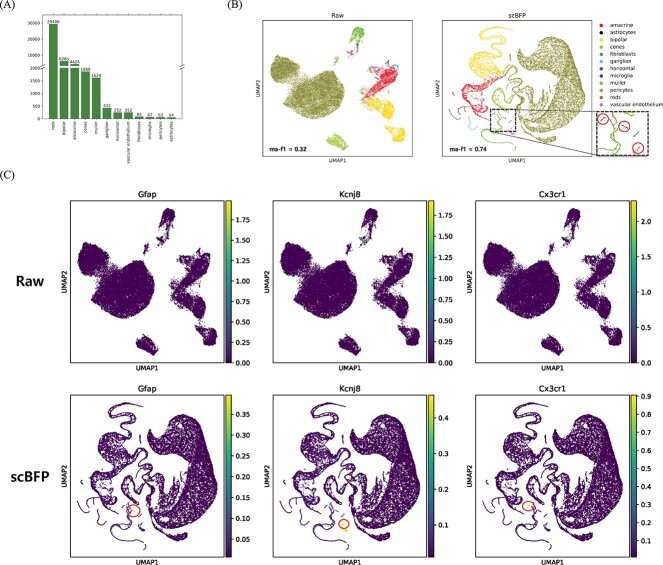
(**A**) Distribution of cell types in the Macosko dataset. (**B**) UMAP visualization comparing raw data and data imputed using the scBFP. (**C**) Visualization of marker gene expression in raw data and data imputed using scBFP, with ‘Gfap,’ ‘Kcnj8’ and ‘Cx3cr1’ serving as marker genes for the ‘astrocytes,’ ‘pericytes’ and ‘microglia’ cell types, respectively.

In addition, we conduct an in-depth analysis of the Macosko dataset to evaluate the effectiveness of scBFP in identifying rare cell types. As shown in Figure 3A, this dataset contains a severe long-tail distribution [22], with the most minority cell type, ‘astrocytes’, consisting of only 54 cells, in contrast to the abundant ‘rods’ type cells, which total 29 400. We visualize the two-dimensional UMAP [23] of the raw counts and the counts imputed via scBFP with the macro-F1 score that is a widely-used metric to evaluate the model’s ability to capture tail cell types in the imbalanced dataset in Figure 3B, and also report the those of baselines in Supplementary Figure S1.

Through these results, we note that the majority of cell types exhibit clear separation based on raw count data alone. However, minority cell types such as ‘astrocytes’, ‘pericytes’ and ‘microglia’ do not separate effectively, and other baseline methods also fall short of capturing these minority cell types, resulting in low macro-F1 performance due to their limited information content. In contrast, scBFP successfully separates minority cell types by capturing inherent data relationships. To further elucidate the ability of scBFP to capture rare cell types, we visualize the expression levels of well-known marker genes, namely ‘Gfap’ for astrocytes, ‘Kcnj8’ for pericytes and ‘Cx3cr1’ for microglia, from data before and after imputed by scBFP in Figure 3C. We also provide this information for the baseline methods in Supplementary Figure S2. Our findings reveal that while the expression levels in raw counts and counts imputed by baselines exhibit relatively high values, they are challenging to differentiate as they are intermingled with cells of other types. In contrast, the imputed data by scBFP effectively captures rare cell types by leveraging marker gene information, as it shows high expression levels and minimal overlap with cells belonging to other cell types. Additionally, we present violin plots illustrating the expression levels of the aforementioned marker genes and other widely recognized marker genes in the data before and after imputation in Supplementary Figure S3. It also demonstrates that our proposed approach, scBFP enhances the signal of marker genes. This enhancement leverages the inherent relationship information present in the original scRNA-seq data. It capitalizes on a property that does not rely on the initially sparse and noisy matrix but rather improves relationships within a ‘warmed-up’ imputed matrix.

### scBFP helps to detect differentially expressed genes

To further emphasize the ability of scBFP to enhance gene-level downstream analysis by amplifying data signals, we conduct an evaluation comparing the impact of scBFP with baseline methods in the task of detecting differentially expressed genes. In this comparison, we utilize a dataset containing both bulk and scRNA-seq data and assess the overlap of DEGs identified using bulk data and scRNA-seq data, treating the outcomes of bulk data as ‘gold standard’ following the experiments setting on previous works [7, 24, 25]. Specifically, we conduct experiments using the Encyclopedia of DNA Elements (ENCODE) samples [26] as input for bulk data and a combined count matrix incorporating five Fluidigm-based ENCODE cell lines [27] for scRNA-seq data. This dataset contains 58 and 362 samples of bulk RNA-seq and scRNA-seq samples, respectively, with five cell types (i.e. ‘A549’, ‘GM12878’, ‘H1-hESC’, ‘IMR90’ and ‘K562’). A more detailed description of these data is provided in the Supplementary Notes 1.2.2.

We utilize the MAST tool [28], a parametric model specifically designed for single-cell data, to detect differentially expressed genes (DEGs). This choice is well-suited for our analysis as it can effectively handle both count and normalized input data. To evaluate DEG detection performance, we identify the top 10, 20,... and 100 DEGs from scRNA-seq data and measure their overlap with DEGs obtained from bulk RNA-seq. The average overlap scores are computed and serve as our primary performance metric. By doing this, we report the overlap scores for all pairs of cell types in Supplementary Figure S4a and present the average of overlap scores across all cell type pairs in Supplementary Figure S4b. These results show that the DEG detection outcomes obtained from the data imputed by scBFP exhibit a higher concordance with DEGs identified through bulk scRNA-seq when compared to those obtained using both raw scRNA-seq data and data imputed by baseline methods. Moreover, it shows robust performance by consistently outperforming raw data for all ten pairs of cell types. Based on these observations, we demonstrate that scBFP enhances the differential gene expression signal by effectively reducing noise in scRNA-seq data while preserving biological variability.

### scBFP effectively recover dropout values

**Figure 4:**
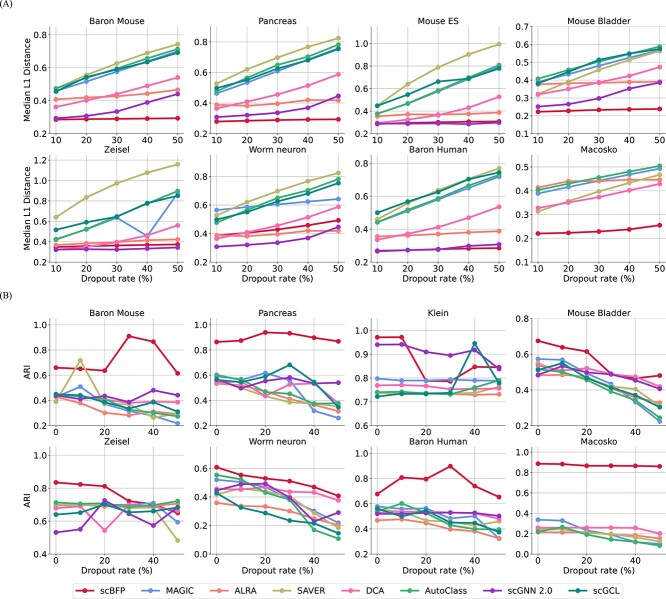
Performance comparison between scBFP and other baselines across eight scRNA-seq datasets. (**A**) and (**B**) Report the dropout recovery and clustering performance under varying dropout rates, respectively.

We assess the capability of scBFP to recover dropout values, a crucial aspect for imputation methods. To verify the robustness of scBFP against dropout phenomena, we conduct experiments by masking a number of non-zero values to zero and measure the difference between masked gene expression values and imputed ones, following the experiments setting of scziDesk [29] and scGNN [10]. Specifically, we quantify the difference between the masked and imputed values using commonly employed metrics, namely the median L1 distance and root mean squared error (RMSE), and report them in Figure 4A and Supplementary Figure S5, respectively. Through these results, we demonstrate that scBFP has the ability to robustly recover the original values even on the high dropout rate. Additionally, our experiments confirm the robustness of scGNN under various dropout rates, verifying the importance of leveraging intercellular relationship information for accurate imputation in noisy scenarios. In contrast, the performance of MAGIC and scGCL, both of which are other graph-based imputation methods, show significant deterioration under the influence of dropout phenomena. We argue that it is since while scGNN iteratively updates the graph structure, MAGIC and scGCL rely on the initial graph derived from raw count data [2]. This observation highlights the crucial role of enhancing graph structure also utilized in scBFP.

Moreover, we also report the clustering performance using this imputed output to ascertain the robustness of scBFP regarding the challenging dropout phenomena frequently encountered in the scRNA-seq domain. As shown in Figure 4B, we verify that scBFP also generally shows superior clustering performance under various dropout rates compared to other baseline methods across various dropout rates, as indicated by the ARI. This performance improvement is attributed to the effective recovery of dropout values, which reduces noise caused by dropout events and subsequently leads to improved clustering accuracy. Additionally, scGNN also exhibits high performance, reinforcing the importance of capturing relationship information with enhanced structural characteristics. Further results, including additional clustering metrics (NMI and CA), can be found in Supplementary Figure S6.

### scBFP enriches relevant genes in lung cancer data

In the above sections, we demonstrate the effectiveness of scBFP in the computational perspective. To check whether scBFP can lead to biologically interpretable insights, we conduct an in-depth analysis on the single-cell lung carcinoid tumor dataset [30]. To this end, we conduct an enrichment analysis using both raw data and data imputed by scBFP and check the difference between them. Our analysis targets epithelial cells [31] because pulmonary carcinoid tumors are neuroendocrine epithelial neoplasms. Specifically, based on absolute fold change $\geq $ 1.25 and false discovery rate (FDR) $\leq $ 0.05 from MAST, 408 and 989 DEGs between epithelial cells of lung carcinoid tissues and that of normal tissues are identified from the raw matrix and the data imputed by scBFP, respectively.

**Figure 5:**
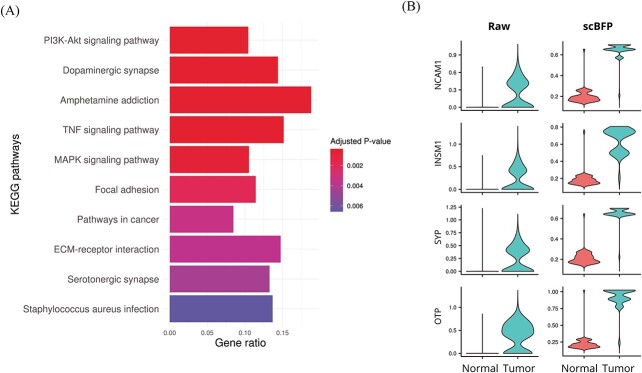
Differential gene analysis between lung carcinoid tumors normal lung tissues. (**A**) Uniquely enriched KEGG pathways from the data imputed by scBFP, compared to that from raw values. The top 10 pathways are displayed, ordered by their adjusted *P*-values. (**B**) Expression-level of scBFP with four markers for lung carcinoid: ‘NCAM1’, ‘INSM1’, ‘SYP’ and ‘OTP’.

In comparison to the DEGs identified using the raw matrix, those derived from scBFP show a clear enrichment in cancer-relevant KEGG pathways [32], as illustrated in Figure 5A, Supplementary Table S4 and S5, which report the top 10 different pathways, and all enriched pathways in the DEGs. Additionally, the ‘PI3K/Akt signaling pathway’, known to be dysregulated in most cancer types, including lung carcinoids, and plays a role in promoting tumor cell growth and neuroendocrine hormone secretion [33, 34], is uniquely enriched. ‘Tumor necrosis factor (TNF) signaling pathway’ is involved in various metabolisms as inflammation, and contributes to cancer progression and metastasis [35, 36]. The ‘MAPK signaling’, which regulates cell proliferation and differentiation, is also one of the representative cancer-associated pathways [37]. Furthermore, the dynamics of ‘focal adhesion’ and ‘ECM-receptor interaction’ can be altered in various types of tumors as carcinoids [38, 39].

Moreover, we also perform the Gene Ontology (GO) enrichment analysis [40, 41] with both raw and data imputed by scBFP and we report the outcomes on Supplementary Table S6 and S7, respectively. We observe that the GO terms identified from the DEGs resulting from scBFP are consistently associated with lung cancer, demonstrating the biological interpretability of scBFP. Specifically, The top three enriched terms in DEGs from scBFP are ‘cytoplasmic translation’ (GO:0002181), ‘negative regulation of apoptotic process’ (GO:0043066), and ‘regulation of cell population proliferation’ (GO:0042127), which are apparently relevant to tumors which require active metabolism, inhibition of apoptosis, and rapid mitosis [42–44]. Other terms such as regulation of ‘ERK1 and ERK2 Cascade’ (GO:0070372) and ‘regulation of MAPK cascade’ (GO:0043408) are reported to be dysregulated by genomic mutations in lung carcinoid tumors [45]. To further ensure consistency of key expression patterns, we compare expression values of four markers of lung carcinoid tumors: NCAM1 (CD35), INSM1, SYP (synaptophysin), and OTP (orthopedia homeobox protein) [46–49]. As depicted in Figure 5(B), the raw data from tumors have a notable amount of missing values (NCAM1: 50%, INSM1: 53%, SYP: 48%, OTP: 30%). scBFP can successfully impute these kinds of data, discriminating the values of normal tissues and tumors. scBFP replaces zeros of raw expression from normal tissues, which are the majority, properly. It is important to note that while classifying and accurately imputing false zeros in normal tissues presents a significant challenge, scBFP effectively imputes such zeros, thereby aiding in differential gene expression analysis.

### scBFP is suitable for high-throughput scRNA-seq data

**Figure 6:**
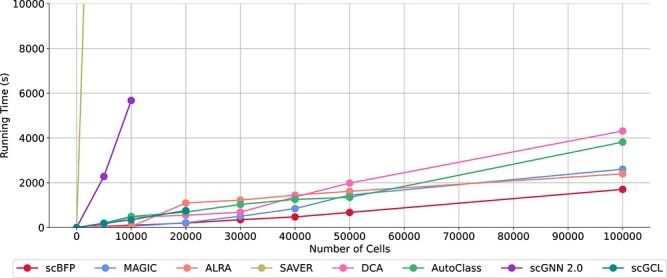
Running time comparison of scBFP and baselines across various number of cells with 10 000 genes.

Given the recent progress in next-generation sequencing technologies, the capacity for scalability in handling high-throughput data is crucial. To evaluate the scalability of scBFP with different cell counts, we generated data ranging from 5K to 100K cells, while maintaining a constant number of 10 000 genes, using the SymSim library [50]. Figure 6 illustrates that the running time of scBFP does not increase exponentially with the number of cells, demonstrating its scalability up to 100k cells. This is attributed to its feature-wise batch training approach, in which the number of genes involved in feature propagation and the number of cells involved in diffusion are determined by the batch size. This is due to the independence of feature channels from the cell and gene perspectives, respectively. In contrast, other graph-based deep-learning methods, such as scGNN and scGCL, are unable to process data with more than 20k to 30k cells due to memory limitations while computing matrix multiplication between feature matrix and adjacency matrix. More details regarding the memory complexity of the baselines can be found in Supplementary Table S8. These findings confirm that scBFP is capable of handling large-scale scRNA-seq datasets, demonstrating its practical applicability in real-world scenarios.

## CONCLUSION AND DISCUSSION

In this study, we present scBFP, a novel method designed to impute the dropout phenomenon in scRNA-seq data while simultaneously addressing downstream tasks. Our approach begins with the gene–cell matrix, imputing zero values from a gene-wise perspective before diffusing both zero and non-zero values through a ‘warmed-up’ matrix in a sequential manner. This bi-level consideration of gene and cell perspectives enables scBFP to effectively recover dropout events, enriching relevant genes and enhancing performance in downstream tasks such as clustering and identifying differentially expressed genes at both cell and gene levels. Additionally, scBFP’s scalability and straightforward implementation hold promise for broader applicability in areas where sparsity is a challenge, such as single-cell Assay for Transposase-Accessible Chromatin using sequencing (scATAC-seq), as demonstrated in Supplementary Figure S7. Moreover, comprehensive ablation studies regarding the two-step approach, diffusion strategies, graph structure and warm-up step of scBFP are provided in Supplementary Notes 1.5.

Moreover, an important consideration that warrants discussion is the treatment of biological zeros, i.e. true zeros. In our methodology, zeros are treated as dropout events requiring imputation. Yet, in reality, biological zeros do exist, and their impact is significant, as noted by [51]. To assess the impact of true zeros, we conducted experiments using a simulation dataset generated by Symsim [50], which allows manual control and analysis of true zeros. First, our analysis delves into how our method achieves favorable outcomes without distinguishing between true and false zeros. Notably, our comparison of the imputed values at true and false zero indices across varying dropout rates in Supplementary Figure S8a reveals that true zeros tend to have smaller imputed values than false zeros. This aligns with the ultimate objective of scRNA-seq imputation: to accurately recover values at false zero indices. This pattern is attributable to the underlying graph structure and diffusion process, where neighboring values of true zeros are smaller than those of false zeros, leading to a pronounced disparity in final imputed values (Supplementary Figure S8b).

Additionally, to verify the impact of true zeros on relevant downstream tasks, we conducted a cell clustering task on both a simulated dataset from Sysmsim and a real dataset. For the real dataset, direct access to true zeros is not feasible, so we approximated the indices of true zeros using the state-of-the-art method, ALRA and illustrated its results in Supplementary Figure S9. We compared scBFP with its variants, specifically focusing on the retention of true zeros as zeros from the initial matrix (input for gene-wise feature propagation) and from the ‘warmed-up’ matrix (input for cell-wise diffusion). Intriguingly, scBFP showed enhanced harmony with the retention of true zeros in the ‘warmed-up’ matrix rather than in the initial matrix. This suggests that during gene-wise feature propagation, facilitating message-passing among zero values is advantageous compared to a one-way message reception from non-zero values. However, when true zeros are retained through ALRA approximation, scBFP did not align as effectively in both simulated and real datasets compared to scenarios with ground truth in simulation, exhibiting similar or marginally lower performance than the original approach. This suggests that the challenge lies in accurately approximating true zeros using ALRA, highlighting a potential area for refinement in real dataset applications. In summary, the incorporation of true zeros presents a notable challenge in real datasets as opposed to simulated ones. Our results indicate that preserving true zeros through the diffusion process does not markedly enhance performance, particularly when considering the added computational burden. Thus, treating zeros as imputation indices in our current approach seems both practical and beneficial under these conditions.

**Key Points**

- We present a novel framework that imputes scRNA-seq data mitigating the risk of propagating false zero values and effectively utilizes both gene–gene and cell–cell relationships.
- Our extensive experiments show that scBFP consistently achieved better performance on various downstream tasks compared to the state-of-the-art models.
- Case on Macosko data shows that scBFP assists in identifying rare cell types.
- In the case of lung cancer scRNA-seq data, scBFP can assist in extracting biologically meaningful insights by enriching relevant genes, showcasing its potential applicability.
- scBFP exhibits scalability, making it well-suited for processing high-throughput scRNA-seq data.

## Supplementary Material

Supplementary_Data_bbae209

## Data Availability

All datasets used in this study are publicly available. Specifically, we obtained the following datasets from the Gene Expression Omnibus (GEO) database: GSE84133 (the Baron data), GSE65525 (the Klein data), GSE60361 (the Zeisel data), GSE63473 (the Macosko data), GSE81861 (the Encode cell lines), GSE196303 (the lung carcinoid tumors), GSE100033 (the forebrain data) and GSE65360 (the insilico data). Additionally, we obtained other datasets from links provided by the respective authors. The pancreas dataset can be accessed at https://ndownloader.figshare.com/files/36086813, the mouse bladder dataset at https://figshare.com/s/865e694ad06d5857db4b and the worm neuron cells dataset at http://waterston.gs.washington.edu/sci_RNA_seq_gene_count_data/Cao_et_al_2017_vignette.RData. Symsim dataset can be publicly accessed through https://github.com/YosefLab/SymSim. We used the recommended setting for UMI parameters ($\alpha $ = 0.04, $\mathit{MaxAmpBias}$ = 0.1, $\mathit{Depth}$ = 5e5) and manually set $\sigma $ = 0.8 to control the heterogeneity across populations and $\mathit{minpopsize}$=10, across a total of five cell types while generating datasets comprising 3000 cells and 2000 genes.

## CODE AVAILABILITY

An implementation of scBFP is publicly available at https://github.com/Junseok0207/scBFP.

## Author Biographies

**Junseok Lee** is researcher at KAIST. His research interest lies in applied machine learning, emphasizing data relationships for analyzing single-cell RNA sequencing data in Bioinformatics.

**Sukwon Yun** is researcher at UNC Chapel Hill. His research interest focuses on leveraging graph structures in Medical and Computational Biology, specifically in single-cell RNA sequencing and Tumor Biology in AI4Science.

**Yeongmin Kim** is researcher at KAIST. His research interest is applying mathematics to biology to extract insights from graph-structured biological data.

**Tianlong Chen** is a postdoctoral researcher at MIT and a member of the Broad Institute of MIT and Harvard. His research interest focuses on building efficient learning systems, emphasizing sparsity, optimization, graph learning, and interdisciplinary scientific challenges.

**Manolis Kellis** is a professor at MIT and a member of the Broad Institute of MIT and Harvard. His research interest focuses on genomics, epigenomics and integrative analysis in *Drosophila* and the NIH Epigenome.

**Chanyoung Park** is a professor at KAIST. His research interest focuses on Data-Centric and Model-Centric AI, extracting insights from diverse data for AI solutions in science, language models and medicine.
