# Supplementary material for: Single-cell RNA sequencing data imputation using bi-level feature propagation
Source: Brief Bioinform. 2024 May 5;25(3):bbae209. doi: 10.1093/bib/bbae209 (PMC11070731; doi:10.1093/bib/bbae209)
Supplement: Supplementary_Data_bbae209 [file supplementary_data_bbae209.pdf]

Supplementary Materials - Single-cell RNA sequencing data imputation using bi-level feature propagation

1 Supplementary Notes

1.1 Implementation detail

scBFP is implemented in Python 3 (version 3.8.8) using PyTorch (https://pytorch.org/) packages. For all experiments all the hyper-parameters are fixed to default values and shared across all the datasets. The number of neighbors in gene-gene and cell-cell graph are set to 10 and number of iterations in gene-wise and cell-wise are set to 10 and 40, respectively. All the experiments are conducted on Intel Xeon Silver 4210R CPU and NVIDIA GeForce RTX 3090 (24GB).

1.2 Experiment settings

1.2.1 Clustering analysis

We employ eight real scRNA-seq datasets (i.e., Baron Mouse [1], Pancreas [2], Klein [3], Mouse bladder cells [4], Zeisel [5], Worm neuron cells [6], Baron Human [1], and Macosko [7] dataset.) that provide gold-standard cell-type information to assess the agreement between predicted clusters and the ground truth. To ensure data quality for subsequent imputation and downstream analysis, we perform minimal quality control (QC) utilizing SCANPY [8]. Given an input gene expression count matrix X_{count}, we filter out the cells and genes with no expression values. Following this step, we calculate the library size l_i individually for each cell i, and subsequently, we normalize the expression values of each cell by dividing them by their respective library sizes and scaling by the median library size. Finally, we apply $log(x + 1)$ transform for all elements in the matrix and obtain the initial gene-cell matrix X for further analysis.

1.2.2 Differentially Expressed Genes analysis

We assess the differential gene expression detection capabilities of scBFP and baseline methods using a dataset that combines bulk and scRNA-seq data. Specifically, we incorporate bulk RNA-seq data from the ENCODE project [9],

consisting of 7 'A549' samples, 11 'GM12878' samples, 8 'H1-hESC' samples, 5 'IMR90' samples, and 27 'K562' samples. The scRNA-seq data, generated using the SMARTer full-length method [10] with the Fluidigm C1 protocol [11], includes five cell lines, comprising a total of 74 'A549' cells, 96 'GM12878' cells, 96 'H1-hESC' cells, 23 'IMR90' cells, and 73 'K562' cells.

In this dataset, we perform normalization to ensure that each cell has the same total count as the median library size and apply a $log(x+1)$ transform, as carried out in the clustering analysis. Subsequently, after conducting imputation using both scBFP and baselines, we proceed to identify differentially expressed genes (DEGs) using the MAST tool [12]. We then adjust the p-values using the Benjamini-Hochberg [13] false discovery rate (FDR) method. To assess the performance, we rank the DEGs based on the adjusted p-values calculated and compute the overlap proportions for the top 10, 20, ..., and 100 DEGs with the bulk RNA-seq DEGs. Finally, we calculate the average of these overlap proportions across all 10 pairs of cell types for the evaluation metric.

1.2.3 Case study of the lung tumor dataset

We filter in cells with several criteria for QC: number of UMIs per cell ≥ 1000, number of genes detected per cell ≥ 500, log10(number of genes detected per UMIs) ≥ 0.8, mitochondrial proportion $\leq 20\%$. We utilize a corrected count for sequencing depth with scTransform [14], then preprocess the matrix with the same steps in the clustering analysis to acquire the initial matrix X. After imputation through scBFP, we identify DEGs between epithelial cells from normal lung tissues and from lung carcinoid tumor samples. Specifically, DEGs are determined based on absolute fold change ≥ 1.25 and false discover rate ≤ 0.05 from MAST, implemented in FindMarkers from Seurat V4 [15]. To investigate functions of the DEGs, gene ontology (GO) [16, 17] and KEGG enrichment [18] analysis are performed via enrichR [19].

1.2.4 ATAC-seq data analysis

In our study, utilizing the cell-by-peak (regions) matrix derived from scATAC-seq data, we preserved the binary nature of scATAC-seq by converting non-zero float values to 1 while retaining zeros as is. Similar to our approach in scRNA-seq analysis, we then implemented minimal quality control using SCANPY. This involved filtering out cells and peaks that exhibited no expression values. Subsequently, we selected 2,000 highly variable peaks, focusing on these specific regions for both the dropout recovery task and the cell clustering task in the Forebrain and InSilico datasets.

1.3 Baseline methods

The imputation and subsequent clustering performance of scBFP is compared with seven state-of-the-art baseline methods incorporating non-graph-based methods, graph-based methods, and statistical methods.

- Markov Affinity-based Graph Imputation of Cells (MAGIC) [20] utilizes Markov chain-based diffusion to smooth and impute single-cell RNA-seq data, enhancing data structure by denoising and revealing relationships between cells.
- Adaptive Low-Rank Approximation (ALRA) [21] reduces noise by identifying biologically true zeros and retaining significant singular values and vectors in scRNA-seq data, leading to effective data reconstruction and denoising.
- Single-cell Analysis Via Expression Recovery (SAVER) [22] employs a Bayesian approach to estimate true gene expression levels, improving the accuracy of data by reducing technical noise in single-cell RNA-seq data.
- Deep Count Autoencoder (DCA) [23] uses a deep learning-based autoencoder to denoise scRNA-seq data, leveraging neural networks to capture complex, non-linear relationships in the data.
- AutoClass [24] is a classic unsupervised classification algorithm that can be applied to scRNA-seq data for clustering and identifying distinct cell types based on gene expression patterns.
- single-cell Graph Neural Network 2.0 (scGNN 2.0) [25] applies graph neural networks, e.g., Graph Attention Network, to scRNA-seq data, integrating gene-gene interaction graphs to enhance cell type identification and data imputation.
- single-cell Graph Contrastive Learning (scGCL) [26] uses a contrastive learning framework on graph-structured scRNA-seq data, aiming to learn robust and discriminative cell embeddings for better clustering and visualization.

1.4 Evaluation Metric

To evaluate the clustering performance of scBFP and baseline methods, we employ four standard evaluation metrics; ARI, NMI, CA, and F1-score, which are defined as follows:

1) The Adjusted Rand Index (ARI) is an adjustment to the Rand Index (RI), defined as:

$$RI = \frac{a+b}{{}_N C_2} \tag{1}$$

where a represents the number of pairs correctly placed in the same cluster, and b represents the number of pairs correctly identified as not belonging to the same cluster. ARI is calculated as follows:

$$ARI = \frac{RI - E[RI]}{max(RI) - E[RI]} \tag{2}$$

where $E[RI]$ represents the expected value of RI. ARI ranges between -1 and 1, with larger values indicating higher agreement between gold standard cell types and predicted cluster assignments.

2) Normalized Mutual Information (NMI) is a metric used to evaluate clustering quality by quantifying the level of agreement between predicted cluster assignments and gold-standard cell types. Specifically, NMI is derived from information theory principles and measures the mutual information between two distributions, in this case, the ground-truth cell types S and the model's cluster assignment C. NMI is calculated as follows:

$$NMI = \frac{2 \times I(S;C)}{[H(S) + H(C)]} \tag{3}$$

where $I(\cdot,\cdot)$ measures the mutual information between two distributions, and H represents the entropy function. NMI falls within the range of 0.0 to 1.0, with higher values indicating stronger alignment between predicted cluster assignments and gold-standard cell types.

3) Clustering Accuracy (CA) serves as a crucial metric for evaluating clustering performance in a manner analogous to supervised classification. Specifically, CA involves finding the optimal matching function that aligns the predicted cluster assignments with the gold-standard cell types, thereby quantifying their agreement. CA is calculated using the following formula:

$$CA = \max_{m} \frac{\sum_{i=1}^{N} \mathbb{1}_{[s_i = m(c_i)]}}{N} \tag{4}$$

Here, N represents the total number of instances, m denotes the matching function responsible for mapping predicted cluster assignments to gold-standard cell types, while s_i and c_i correspond to the gold-standard cell type and predicted cluster assignment of the i-th cell, respectively.

4) F1-score is a valuable metric, particularly useful in addressing scenarios with imbalanced data. It balances precision and recall, rendering it suitable for binary classification settings. Precision quantifies the accuracy of positive predictions relative to all positive predictions, while recall measures the proportion of correctly identified positive instances out of all actual positives. F1-score is computed as follows:

$$F1\text{-}score = \frac{2(Precision \times Recall)}{Precision + Recall} \tag{5}$$

Furthermore, in the context of assessing clustering performance, we identify the optimal matching function for mapping predicted cluster assignments to the gold-standard cell types. To extend this evaluation to a multi-class setting, we utilize the macro-average approach, referred to as the Macro-F1 Score (ma-F1).

On the other hand, we evaluate the ability of scBFP and baseline methods to recover dropout values using two evaluation metrics: Median L1 Distance and RMSE. These metrics are defined as follows:

1) Median L1 Distance is a metric used to measure the difference between two sets of values, calculating the median of the absolute differences between corresponding elements. Mathematically, the Median L1 Distance between two sets of values $X = \{x_1, x_2, ..., x_n\}$ and $Y = \{y_1, y_2, ..., y_n\}$,which denote the imputed and original values, respectively, can be defined as:

$$Median\ L1\ Distance = median(|x_1 - y_1|, |x_2 - y_2|, ..., |x_n - y_n|) \tag{6}$$

2) Root Mean Square Error (RMSE) is a commonly used metric that quantifies the average root squared difference between predicted and original values, measuring the difference between them. The RMSE between sets of values X and Y is calculated as

$$RMSE(X, Y) = \sqrt{\frac{\sum_{i=1}^{N}(x_i - y_i)^2}{N}} \tag{7}$$

where lower values for both the Median L1 Distance and RMSE indicate smaller differences between the original and imputed values.

1.5 Component analysis of scBFP

We conduct a comprehensive analysis of all components of scBFP, beginning with an assessment of the necessity of the two-step strategies designed to mitigate noise propagation caused by dropout phenomena and to leverage both gene-gene and cell-cell relationships. In Supplementary Figure S10, we directly compare our proposed two-step approach with all potential one-step strategies, which include variations utilizing cell-cell or gene-gene graphs and employing propagation techniques with diffusion (propagating both zero and non-zero values) or feature propagation (retaining only non-zero values). The results indicate that scBFP consistently outperforms other variations, verifying the significance of a two-step strategy.

For a more detailed analysis, we perform ablation experiments by selectively changing either the propagation strategy or graph structures. In Supplementary Figure S11, we compare the performance of scBFP with variations where diffusion is applied in both the first and second steps or feature propagation is conducted in both steps. These results indicate that maintaining the non-zero values in both steps (i.e., w/ replacement $\rightarrow$ w/ replacement) leads to relatively lower performance. It shows that since non-zero values also contain biologically irrelevant signals like amplification bias, removing such noise is crucial. Conversely, when diffusing zero values in the first step (i.e., w/o replacement $\rightarrow$ w/o replacement), we also observe lower performance than scBFP, indicating the potential risk of noise propagation without a warm-up step. Moreover, we

also conduct the ablation experiments regarding the graph structure on Supplementary Figure S12. These observations indicate that propagating information with a gene-gene graph alone can result in poorer performance due to the smoothing of biologically relevant information. However, when the gene-gene graph is utilized in the warm-up step and the cell-cell graph is employed in the subsequent step, it leads to improved performance by providing complementary information. Additionally, we examine whether utilizing the improved graph constructed from the warmed-up matrix yields benefits compared to the graph constructed from the initial matrix. Supplementary Figure S13 illustrates that leveraging the cell-cell graph constructed from the 'warmed-up matrix' generally outperforms the alternative, albeit with slightly lower performance observed on the 'Baron Mouse' and 'Baron Human' datasets. In Supplementary Figure S14, we clarify that this phenomenon is attributed to the fact that the graph constructed from the initial data already exhibits a homophily ratio exceeding 90% in these two datasets, indicating that further enhancement is unnecessary. Based on these findings, we can conclude that our warm-up step effectively enhances the graph structure, leading to a higher homophily ratio and an overall improvement in downstream task performance.

In extending our analysis, we conducted a detailed examination of key parameters: the number of neighboring nodes (k) for constructing gene-gene and cell-cell graphs, and the number of iterations for Gene-wise Feature Propagation and Cell-wise Diffusion, as presented in Supplementary Figure S15. We observed that a small number of neighbors is insufficient, as it limits the potential for message propagation, thereby failing to effectively mitigate dropout phenomena among neighboring nodes. Conversely, an excessively high number of neighbors does not confer substantial benefits compared to a moderate number, such as $k = 10$. This is because an abundance of neighboring nodes and their associated messages can introduce noise, thereby obscuring the discernibility among different cell types. Furthermore, our analysis reveals that when the number of iterations for cell processing is fixed while increasing the number of gene iterations, the imputation task remains robust. This robustness can be attributed to the preservation of the initial state of non-zero values, which maintain their original scale and assist solely in the imputation of neighboring zero values. However, for the cell clustering task, an excessive number of gene iterations does not yield significant advantages, as overly smoothed gene expressions can diminish the distinctiveness of cells, a characteristic that is crucial for effective clustering. In terms of cell iterations, we found that a higher number, such as 40, is advantageous for downstream tasks that require a smoothed cell representation. This smoothed representation, particularly beneficial for clustering tasks, facilitates the grouping of similar cell types. In conclusion, our findings indicate that a moderate number of neighboring nodes (k), coupled with a relatively smaller number of gene iterations and a higher number of cell iterations, proves to be optimal for both dropout recovery and clustering tasks.

2 Supplementary Figures

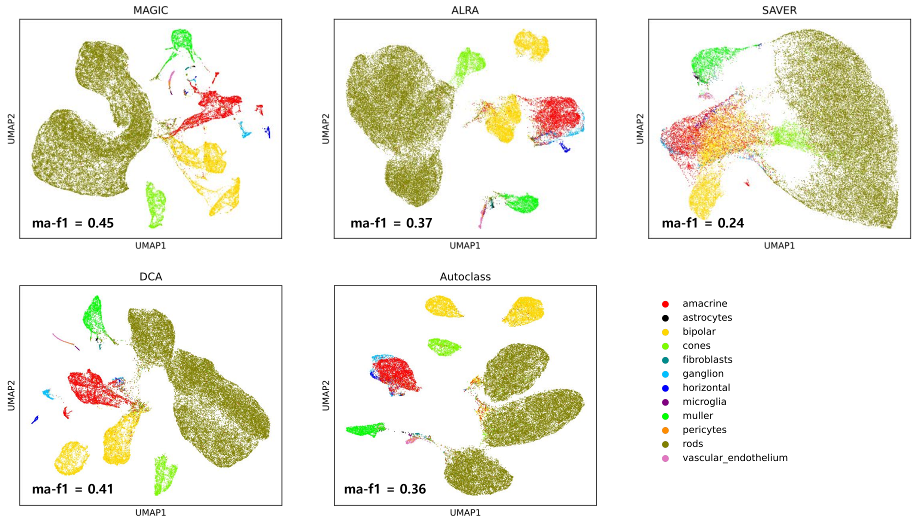

Supplementary Figure S1: UMAP visualization and micro-f1 score performances of baseline methods in Macoksko dataset.

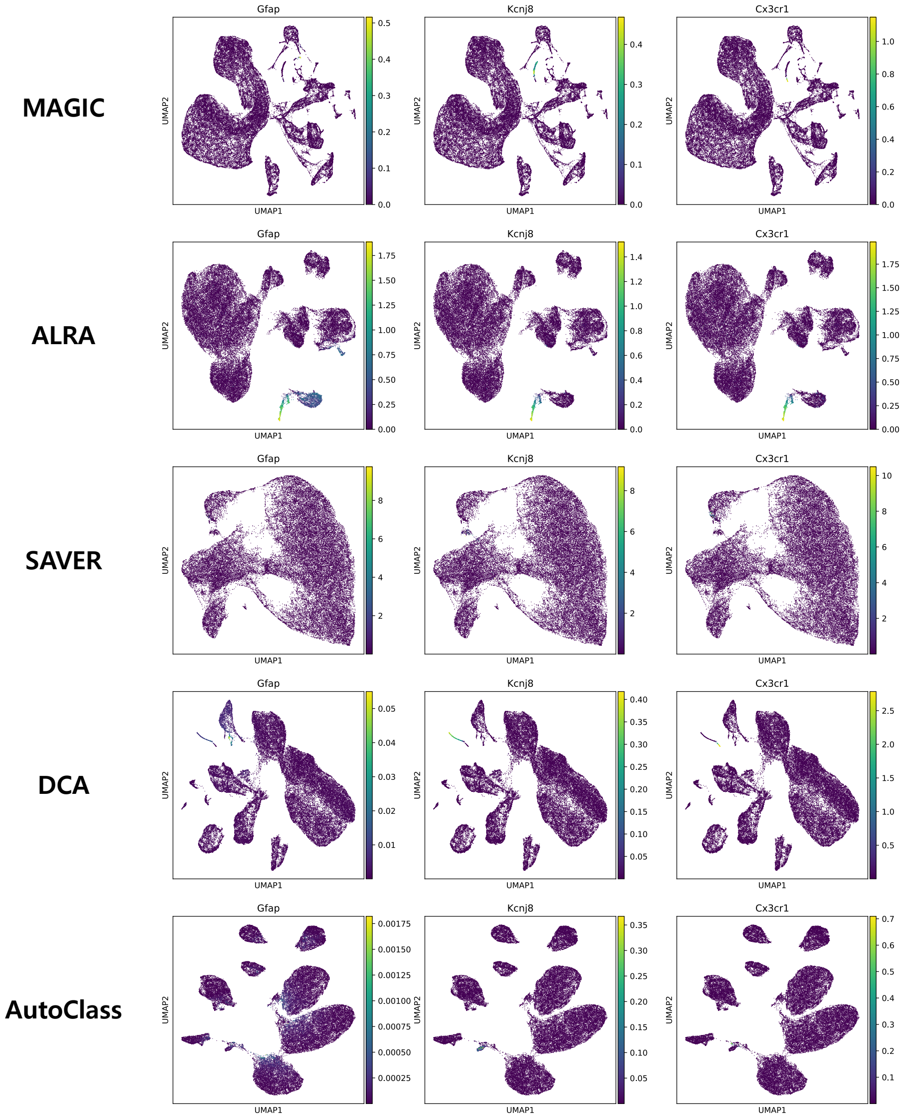

Supplementary Figure S2: Visualization of marker gene expression in data imputed using baseline methods, with 'Gfap,' 'Kcnj8,' and 'Cx3cr1' serving as marker genes for the 'astrocytes,' 'pericytes,' and 'microglia' cell types, respectively.

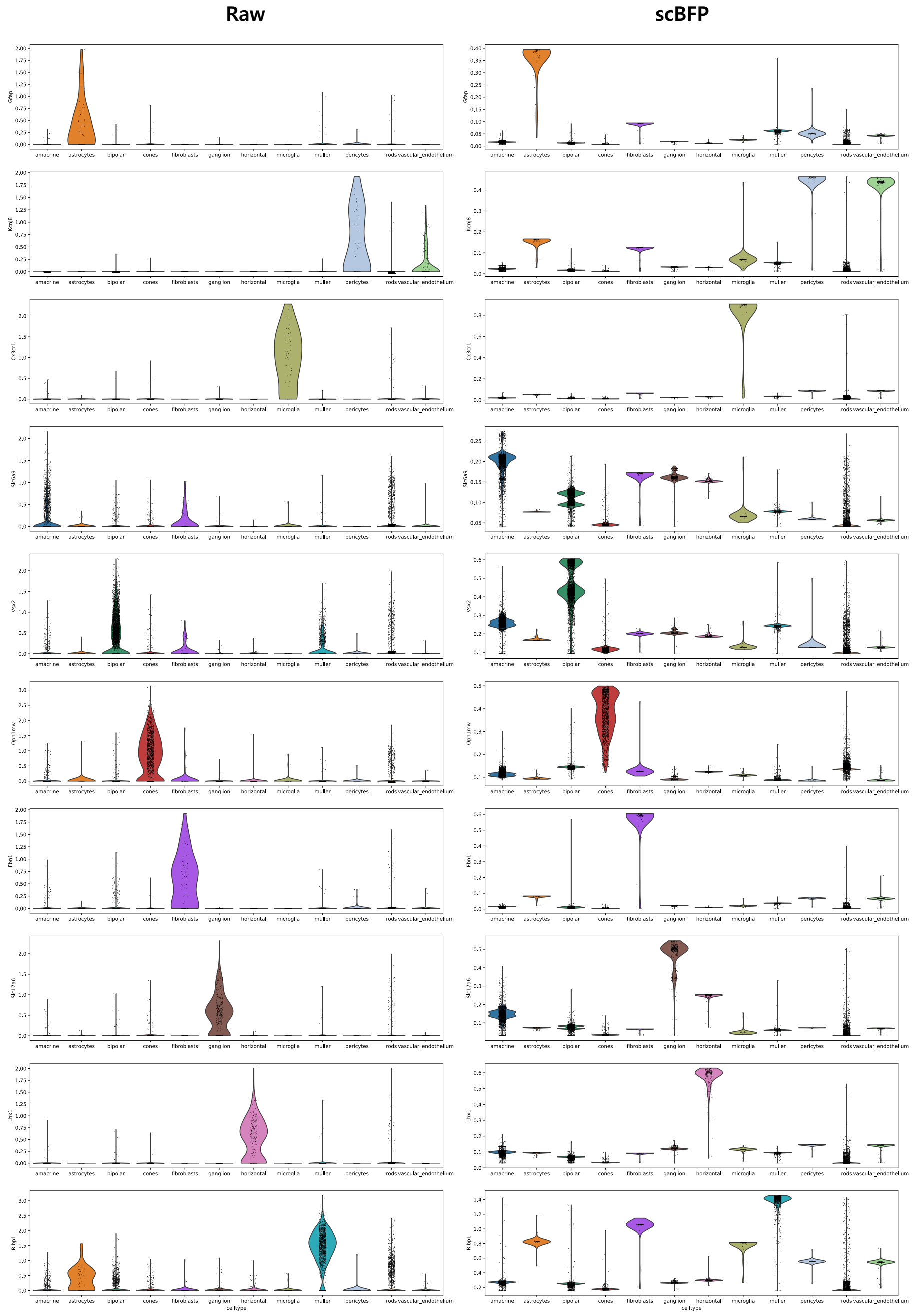

Supplementary Figure S3: Violin plots showing expression levels of marker genes before and after imputation in Macosko data.

(a)

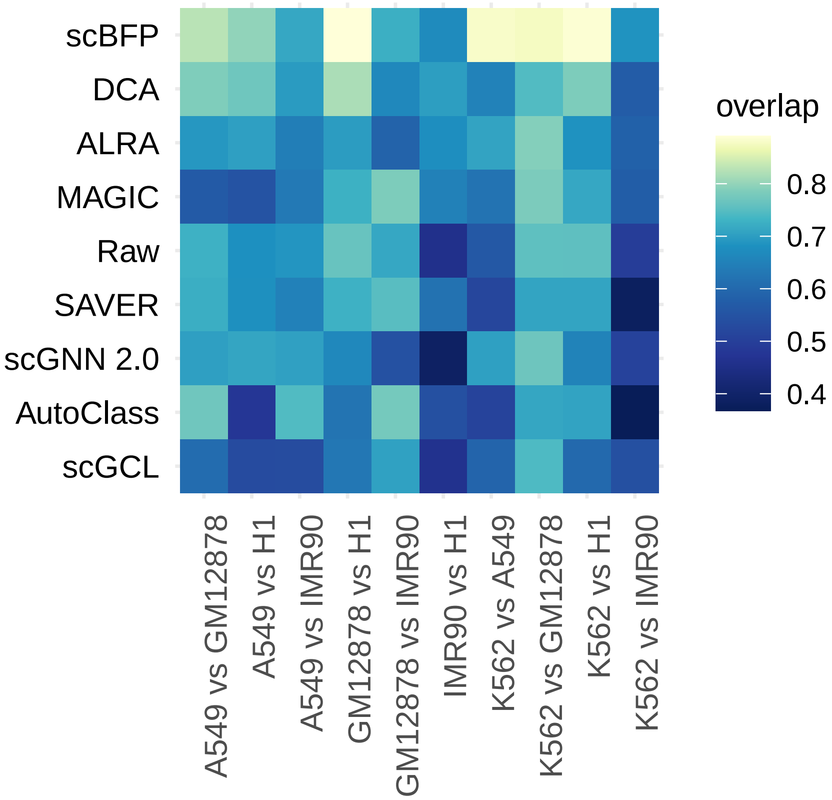

(b)

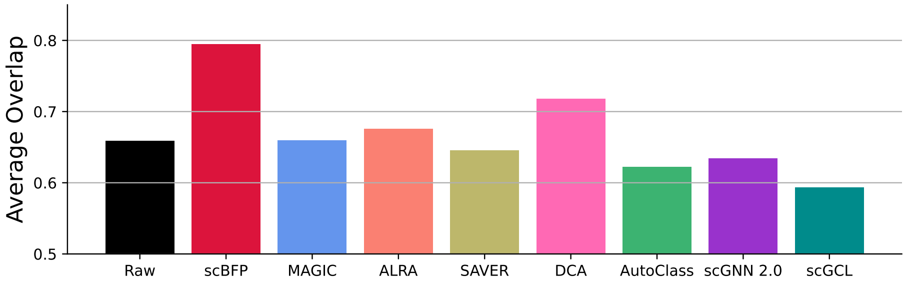

Supplementary Figure S4: Comparison of DEG detection performance between scBFP and baseline methods. (a) Shows the overlap between DEGs identified from bulk and scRNA-seq data for all pairs of cell types. (b) Presents the average overlap ratio across all pairs of cell types.

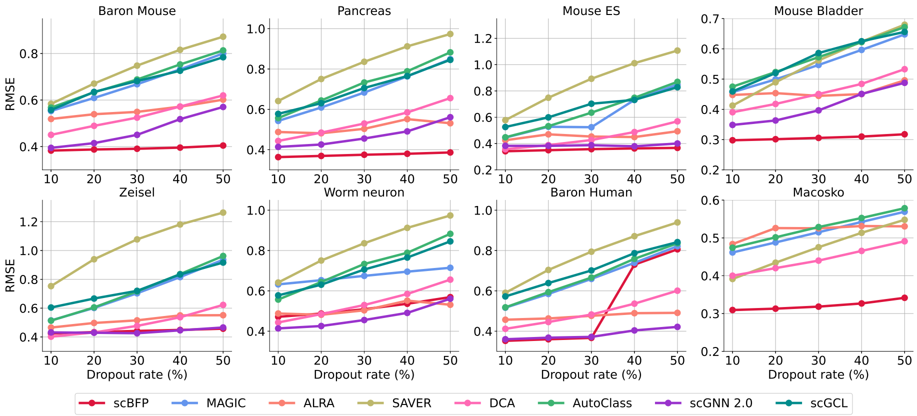

Supplementary Figure S5: Dropout recovery performance comparison between scBFP and other baselines across eight scRNA-seq datasets in terms RMSE.

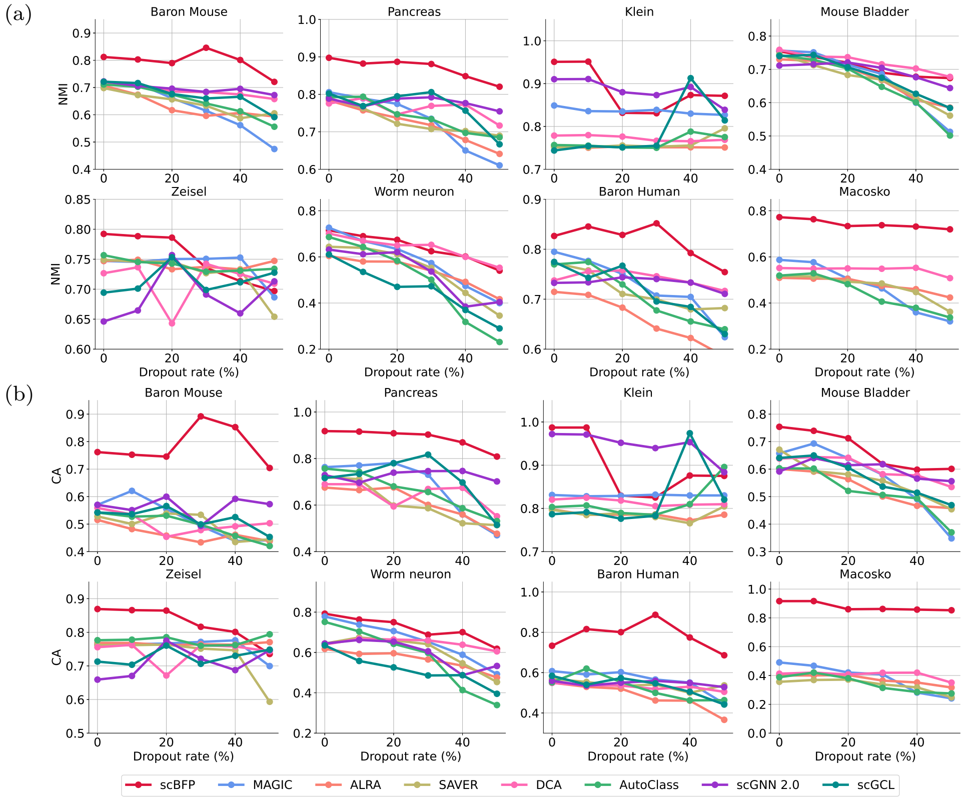

Supplementary Figure S6: Clustering performance comparison between scBFP and other baselines across eight scRNA-seq datasets in terms of NMI and CA.

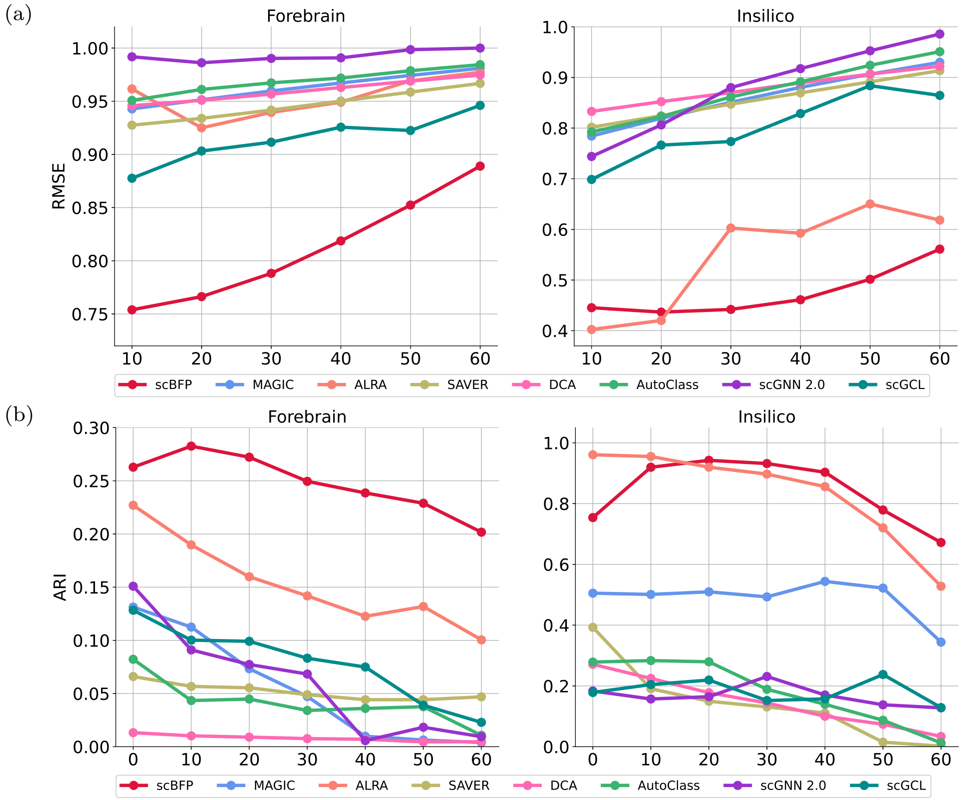

Supplementary Figure S7: Generalization on scATAC-seq domain using Forebrain and InSilico datasets. (a) Peak recovery performance comparison upon various drop rates between scBFP and other baselines in terms of RMSE. (b) Cell clustering performance comparison upon various drop rates between scBFP and other baselines in terms of NMI.

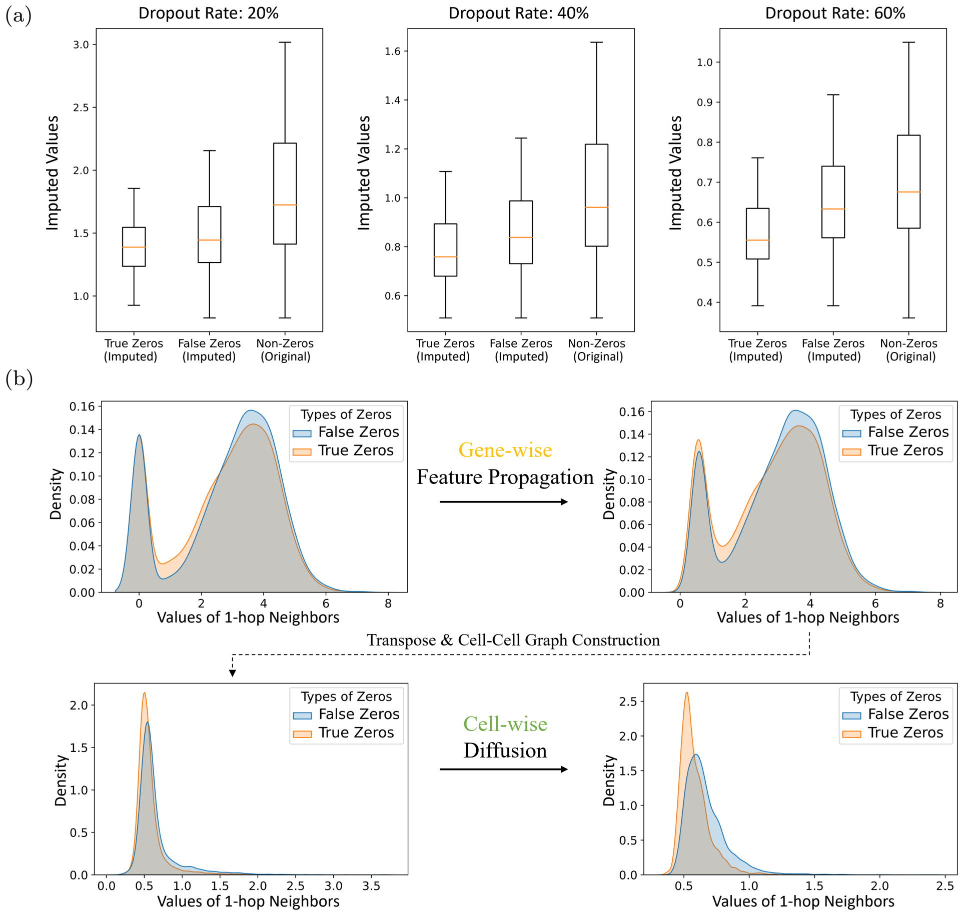

Supplementary Figure S8: Imputed Values within True Zeros and False Zeros using a simulation dataset via Symsim: (a) Box plot of imputed values via scBFP in terms of True Zeros, False Zeros, and Non-Zeros. (b) Distribution plot of randomly sampled 10,000 values of 1-hop neighbors of False Zeros and True Zeros throughout the procedure of the scBFP. A dataset with a 60% dropout rate was used.

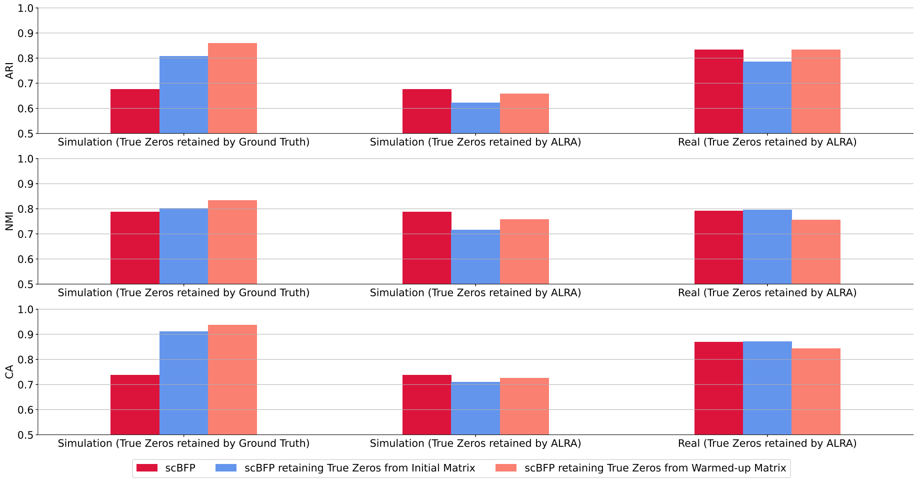

Supplementary Figure S9: Comparison of performance of scBFP and its variants of considering true (approximated) zeros between simulation dataset generated by Symsim with real dataset, Zeisel.

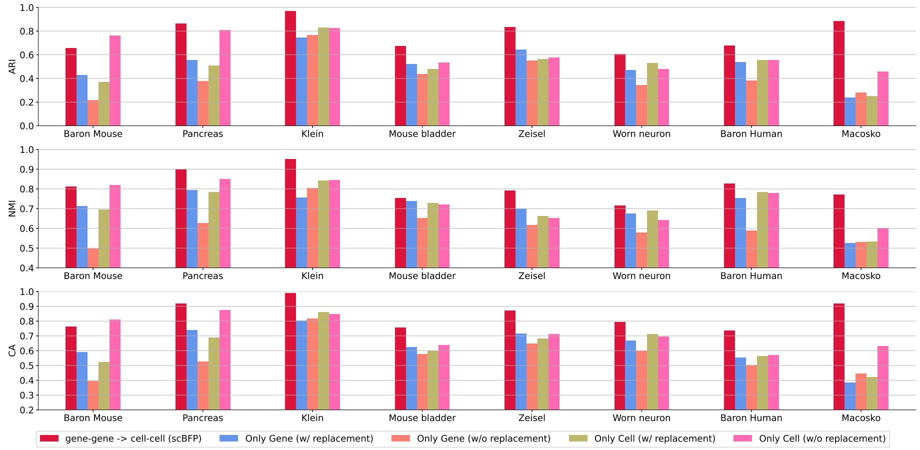

Supplementary Figure S10: Ablation studies on scBFP regarding the necessity of a two-step propagation strategy across eight scRNA-seq datasets.

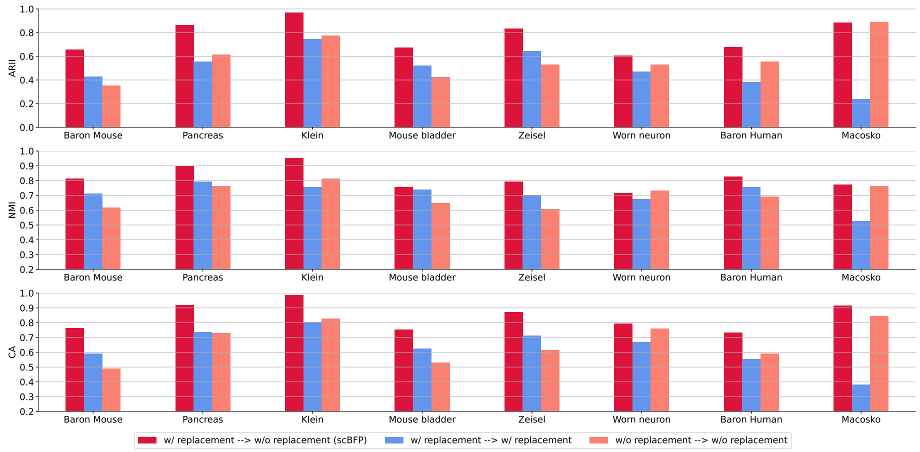

Supplementary Figure S11: Ablation studies on scBFP regarding the diffusion strategies across eight scRNA-seq datasets.

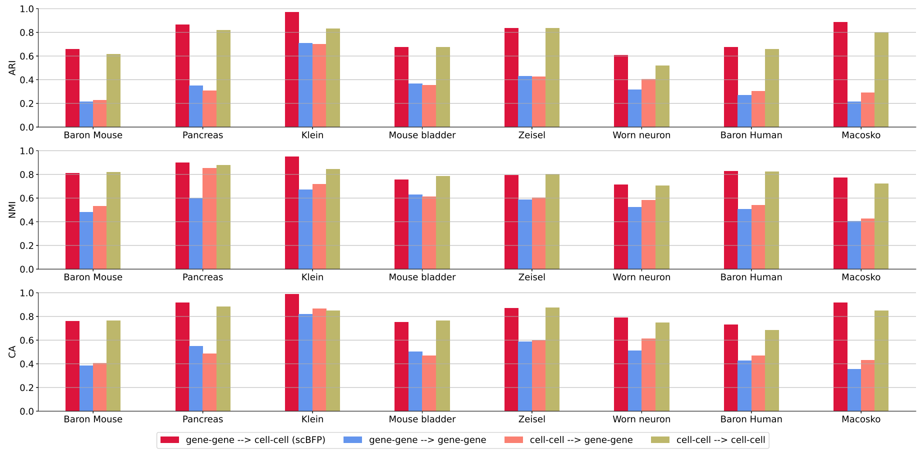

Supplementary Figure S12: Ablation studies on scBFP regarding the graph structure across eight scRNA-seq datasets.

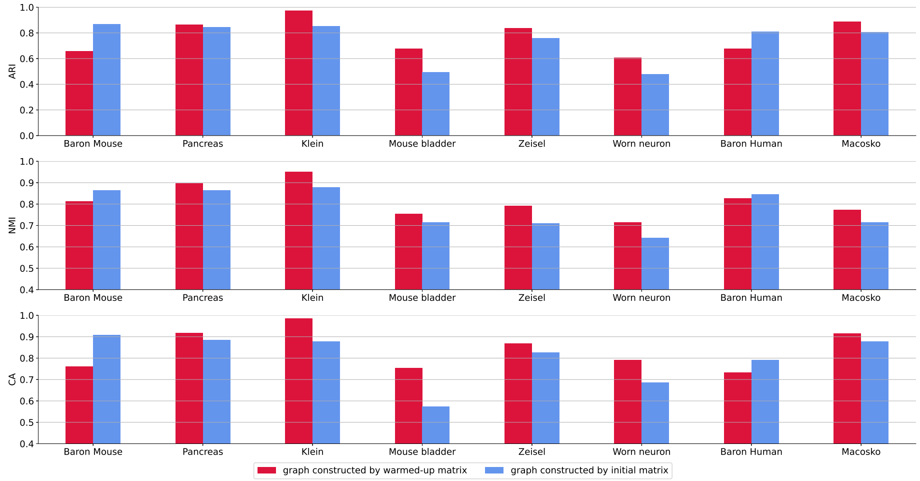

Supplementary Figure S13: Comparison of performance between using graphs constructed by the initial matrix and those constructed with the 'warmed-up' matrix.

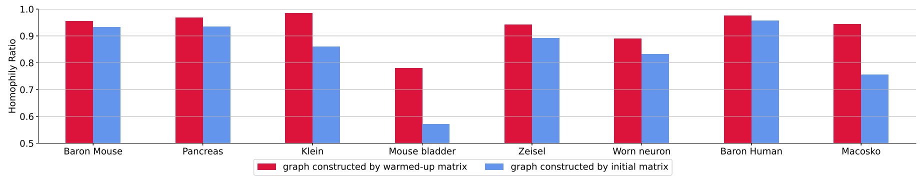

Supplementary Figure S14: Comparison of homophily ratio (i.e., the ratio of edges connected between the nodes with same cell type) between graph constructed by 'warmed-up' matrix and initial raw matrix.

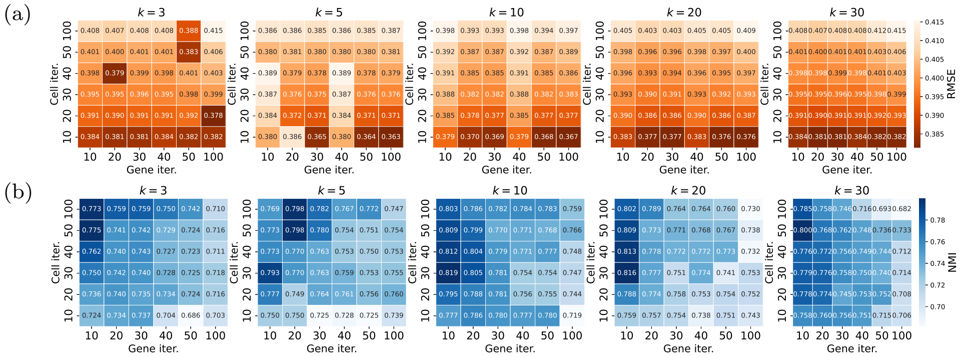

Supplementary Figure S15: Sensitivity analysis on parameters used from scBFP in Baron Mouse dataset. 'k' denotes the number of neighbors in both the gene-gene graph and cell-cell graph, 'Gene iter.' and 'Cell iter.' denote the number of iterations in Gene-wise Feature Propagation and Cell-wise Diffusion, respectively. (a) Dropout recovery task in terms of RMSE (smaller, the better). (b) Cell clustering task in terms of NMI (higher, the better).

3 Supplementary Tables

Data	Sequencing platform	# of Cells	# of Genes	# of cell types
Baron mouse	inDrop	1,886	14,861	13
Pancreas	inDrop	1,937	15,575	14
Mouse ES cells	inDrop	2,717	24,047	4
Mouse bladder cells	Microwell-seq	2,746	19,771	16
Zeisel	STRT-seq UMI	3,005	19,972	7
Worm neuron cells	sci-RNA-seq	4,186	13,488	10
Baron Human	inDrop	8,569	17,499	14
Macosko mouse retina cells	Drop-seq	44,808	23,288	19

Supplementary Table S1: Statistics for scRNA-seq datasets with gold-standard cell types.

Method	Language	Reference
MAGIC	Python	`https://github.com/KrishnaswamyLab/MAGIC`
ALRA	R	`https://github.com/KlugerLab/ALRA`
SAVER	R	`https://github.com/mohuangx/SAVER`
DCA	Python	`https://github.com/theislab/dca`
AutoClass	Python	`https://github.com/datapplab/AutoClass`
scGNN 2.0	Python	`https://github.com/OSU-BMBL/scGNN2.0`
scGCL	Python	`https://github.com/zehaoxiong123/scGCL`

Supplementary Table S2: Link for the source codes of the baseline methods

Metric	MAGIC	ALRA	SAVER	DCA	AutoClass	scGNN 2.0	scGCL
ARI	0.0039	0.0039	0.0039	0.0039	0.0039	0.0078	0.0078
NMI	0.0195	0.0039	0.0039	0.0078	0.0039	0.0078	0.0078
CA	0.0039	0.0039	0.0039	0.0039	0.0039	0.0078	0.0078

Supplementary Table S3: One-sided Wilcoxon signed-rank test results between scBFP and baseline methods. Specifically, we report the p-value to verify whether the clustering performance of scBFP is significantly greater than that of other baselines in terms of ARI, NMI, and CA.

Term	Adjusted p value
Parkinson disease	1.17e-13
Prion disease	7.29e-12
Pathways of neurodegeneration	6.81e-11
Oxidative phosphorylation	2.88e-10
Alzheimer disease	7.09e-10
Coronavirus disease	1.19e-09
Diabetic cardiomyopathy	3.72e-09
Huntington disease	5.23e-09
Amyotrophic lateral sclerosis	1.30e-08
Antigen processing and presentation	2.28e-07
Fluid shear stress and atherosclerosis	3.28e-07
Kaposi sarcoma-associated herpesvirus infection	3.28e-07
Non-alcoholic fatty liver disease	4.17e-07
Protein processing in endoplasmic reticulum	5.16e-07
Ribosome	5.16e-07
Thermogenesis	2.51e-06
Protein export	9.87e-06
IL-17 signaling pathway	1.18e-05
Phagosome	1.93e-05
Legionellosis	4.67e-05
Rheumatoid arthritis	4.80e-05
Viral myocarditis	7.18e-05
Pertussis	1.31e-04
Type I diabetes mellitus	1.52e-04
Lipid and atherosclerosis	2.15e-04
Epithelial cell signaling in Helicobacter pylori infection	2.80e-04
Mineral absorption	3.70e-04
Pathogenic Escherichia coli infection	5.60e-04
Leishmaniasis	6.23e-04
Estrogen signaling pathway	7.13e-04
Tight junction	7.13e-04
Th17 cell differentiation	7.13e-04
Proximal tubule bicarbonate reclamation	8.53e-04
Complement and coagulation cascades	1.29e-03
Salmonella infection	1.29e-03
Cell adhesion molecules	1.49e-03
Cardiac muscle contraction	1.50e-03
Human T-cell leukemia virus 1 infection	1.67e-03
Ferroptosis	3.27e-03
Leukocyte transendothelial migration	4.05e-03
Retrograde endocannabinoid signaling	4.26e-03
Shigellosis	6.35e-03
Cellular senescence	6.82e-03
Cholesterol metabolism	9.85e-03

Supplementary Table S4: Enriched KEGG pathways (Adjusted p value ≤ 0.01) in DEGs identified by MAST in the raw data

Term	Adjusted p value
Coronavirus disease	1.21e-09
IL-17 signaling pathway	6.65e-07
Fluid shear stress and atherosclerosis	7.58e-06
Tight junction	1.81e-05
Legionellosis	2.76e-05
Rheumatoid arthritis	2.76e-05
Complement and coagulation cascades	2.98e-05
Ribosome	3.28e-05
Pertussis	1.23e-04
Antigen processing and presentation	1.55e-04
Prion disease	3.61e-04
PI3K-Akt signaling pathway	3.61e-04
Parkinson disease	3.73e-04
Lipid and atherosclerosis	4.85e-04
Dopaminergic synapse	4.91e-04
Amphetamine addiction	4.91e-04
Kaposi sarcoma-associated herpesvirus infection	4.91e-04
Mineral absorption	4.91e-04
TNF signaling pathway	5.27e-04
MAPK signaling pathway	8.80e-04
Cell adhesion molecules	1.77e-03
Estrogen signaling pathway	1.87e-03
Focal adhesion	1.98e-03
Phagosome	2.21e-03
Pathways of neurodegeneration	2.33e-03
Pathogenic Escherichia coli infection	3.34e-03
Pathways in cancer	3.34e-03
ECM-receptor interaction	3.70e-03
Leishmaniasis	3.70e-03
Serotonergic synapse	4.27e-03
Epithelial cell signaling in Helicobacter pylori infection	5.59e-03
Protein export	6.27e-03
Staphylococcus aureus infection	6.57e-03
Th17 cell differentiation	6.57e-03
Human cytomegalovirus infection	6.57e-03
Type I diabetes mellitus	8.52e-03
AGE-RAGE signaling pathway in diabetic complications	9.90e-03

Supplementary Table S5: Enriched KEGG pathways (Adjusted p value ≤ 0.01) in DEGs identified by MAST in the data imputed by `scBFP`

Term	Adjusted.P.value
Negative Regulation Of Apoptotic Process (GO:0043066)	1.12e-09
Cytoplasmic Translation (GO:0002181)	8.52e-09
Negative Regulation Of Programmed Cell Death (GO:0043069)	1.32e-07
Mitochondrial ATP Synthesis Coupled Electron Transport (GO:0042775)	1.32e-07
Aerobic Electron Transport Chain (GO:0019646)	4.91e-07
Cellular Response To Cadmium Ion (GO:0071276)	4.91e-07
Peptide Biosynthetic Process (GO:0043043)	4.91e-07
Cellular Response To Metal Ion (GO:0071248)	5.46e-07
Response To Unfolded Protein (GO:0006986)	6.03e-07
Cellular Respiration (GO:0045333)	1.32e-06
Proton Motive Force-Driven ATP Synthesis (GO:0015986)	3.45e-06
Response To Cadmium Ion (GO:0046686)	3.51e-06
Regulation Of Apoptotic Process (GO:0042981)	4.02e-06
Macromolecule Biosynthetic Process (GO:0009059)	4.81e-06
Oxidative Phosphorylation (GO:0006119)	4.95e-06
Response To Copper Ion (GO:0046688)	1.07e-05
Aerobic Respiration (GO:0009060)	1.54e-05
Cellular Response To Copper Ion (GO:0071280)	2.95e-05
Cellular Response To Zinc Ion (GO:0071294)	4.72e-05
Translation (GO:0006412)	9.66e-05
Cellular Response To Heat (GO:0034605)	1.12e-04
Negative Regulation Of Intrinsic Apoptotic Signaling Pathway (GO:2001243)	1.38e-04
Response To Zinc Ion (GO:0010043)	1.70e-04
Proton Motive Force-Driven Mitochondrial ATP Synthesis (GO:0042776)	2.05e-04
Gene Expression (GO:0010467)	6.00e-04
Regulation Of Cell Population Proliferation (GO:0042127)	7.62e-04
Mitochondrial Electron Transport, Cytochrome C To Oxygen (GO:0006123)	1.14e-03
Surfactant Homeostasis (GO:0043129)	1.17e-03
Energy Derivation By Oxidation Of Organic Compounds (GO:0015980)	1.38e-03
Epithelial Cell Differentiation (GO:0030855)	1.38e-03
Positive Regulation Of Binding (GO:0051099)	1.45e-03
Mitochondrial Electron Transport, NADH To Ubiquinone (GO:0006120)	1.55e-03
Positive Regulation Of Cyclin-Dependent Protein Kinase Activity (GO:1904031)	2.04e-03
Chaperone Cofactor-Dependent Protein Refolding (GO:0051085)	2.53e-03
Positive Regulation Of Protein Modification Process (GO:0031401)	3.39e-03
Regulation Of Immune Effector Process (GO:0002697)	4.28e-03
Chaperone-Mediated Protein Complex Assembly (GO:0051131)	4.79e-03
Negative Regulation Of Cellular Process (GO:0048523)	4.79e-03
Negative Regulation Of Protein Phosphorylation (GO:0001933)	4.79e-03
Response To Metal Ion (GO:0010038)	4.98e-03
Response To Calcium Ion (GO:0051592)	5.47e-03
Cellular Response To Organic Cyclic Compound (GO:0071407)	5.47e-03
Modulation By Host Of Viral Process (GO:0044788)	5.47e-03
Negative Regulation Of Myeloid Cell Differentiation (GO:0045638)	5.47e-03
Substantia Nigra Development (GO:0021762)	5.63e-03
'De Novo' Post-Translational Protein Folding (GO:0051084)	6.11e-03
Negative Regulation Of Growth (GO:0045926)	6.59e-03
Negative Regulation Of Endoplasmic Reticulum Stress-Induced Intrinsic Apoptotic Signaling Pathway (GO:1902236)	6.92e-03
Positive Regulation Of Cyclin-Dependent Protein Serine/Threonine Kinase Activity (GO:0045737)	6.92e-03
Positive Regulation Of Cell Differentiation (GO:0045597)	8.78e-03
Regulation Of Protein Ubiquitination (GO:0031396)	8.80e-03
Positive Regulation Of Protein Kinase Activity (GO:0045860)	9.07e-03
Regulation Of Tau-Protein Kinase Activity (GO:1902947)	9.07e-03
Negative Regulation Of Response To Endoplasmic Reticulum Stress (GO:1903573)	9.67e-03

Supplementary Table S6: Enriched GO terms (Adjusted p value $\leq$ 0.01) in DEGs identified by MAST in the raw data

Term	Adjusted p value
Cytoplasmic Translation (GO:0002181)	2.15e-06
Negative Regulation Of Apoptotic Process (GO:0043066)	3.01e-05
Regulation Of Cell Population Proliferation (GO:0042127)	3.01e-05
Cellular Response To Metal Ion (GO:0071248)	1.63e-04
Peptide Biosynthetic Process (GO:0043043)	1.64e-04
Positive Regulation Of Cell Population Proliferation (GO:0008284)	1.64e-04
Cellular Response To Cadmium Ion (GO:0071276)	2.32e-04
Response To Unfolded Protein (GO:0006986)	4.16e-04
Generation Of Neurons (GO:0048699)	4.84e-04
Regulation Of ERK1 And ERK2 Cascade (GO:0070372)	4.91e-04
Cellular Response To Copper Ion (GO:0071280)	8.01e-04
Positive Regulation Of Cellular Process (GO:0048522)	8.20e-04
Cellular Response To Zinc Ion (GO:0071294)	8.47e-04
Regulation Of Kinase Activity (GO:0043549)	8.75e-04
Response To Cadmium Ion (GO:0046686)	9.08e-04
Positive Regulation Of Cell Differentiation (GO:0045597)	9.56e-04
Macrophage Chemotaxis (GO:0048246)	1.08e-03
Negative Regulation Of Programmed Cell Death (GO:0043069)	1.19e-03
Negative Regulation Of Intrinsic Apoptotic Signaling Pathway (GO:2001243)	1.27e-03
Regulation Of Exocytosis (GO:0017157)	1.68e-03
Macrophage Migration (GO:1905517)	1.68e-03
Nervous System Development (GO:0007399)	1.72e-03
Positive Regulation Of MAPK Cascade (GO:0043410)	1.72e-03
Macromolecule Biosynthetic Process (GO:0009059)	1.72e-03
Response To Copper Ion (GO:0046688)	1.72e-03
Negative Regulation Of Cellular Process (GO:0048523)	1.80e-03
Response To Zinc Ion (GO:0010043)	2.25e-03
Epithelial Cell Differentiation (GO:0030855)	3.10e-03
Regulation Of Cell Migration (GO:0030334)	3.10e-03
Regulation Of MAPK Cascade (GO:0043408)	3.10e-03
Positive Regulation Of Nitric Oxide Biosynthetic Process (GO:0045429)	3.56e-03
Positive Regulation Of Response To Wounding (GO:1903036)	3.56e-03
Positive Regulation Of Binding (GO:0051099)	3.89e-03
Negative Regulation Of Growth (GO:0045926)	3.89e-03
Negative Regulation Of Signaling (GO:0023057)	4.24e-03
Positive Regulation Of Nitric Oxide Metabolic Process (GO:1904407)	4.24e-03
Negative Regulation Of Protein Phosphorylation (GO:0001933)	4.30e-03
Negative Regulation Of Endoplasmic Reticulum Stress-Induced Intrinsic Apoptotic Signaling Pathway (GO:1902236)	4.42e-03
Regulation Of Protein Phosphorylation (GO:0001932)	4.76e-03
Surfactant Homeostasis (GO:0043129)	5.13e-03
Regulation Of Osteoblast Differentiation (GO:0045667)	5.69e-03
Central Nervous System Development (GO:0007417)	5.94e-03
Epithelium Development (GO:0060429)	5.94e-03
Positive Regulation Of Unsaturated Fatty Acid Biosynthetic Process (GO:2001280)	6.41e-03
Regulation Of Nitric Oxide Biosynthetic Process (GO:0045428)	7.03e-03
Retina Homeostasis (GO:0001895)	7.03e-03
Positive Regulation Of Cell Motility (GO:2000147)	7.43e-03
Antibacterial Humoral Response (GO:0019731)	8.43e-03
Neuron Differentiation (GO:0030182)	9.30e-03
Synaptic Vesicle Exocytosis (GO:0016079)	9.59e-03
Visual System Development (GO:0150063)	9.59e-03

Supplementary Table S7: Enriched GO terms (Adjusted p value ≤ 0.01) in DEGs identified by MAST in the data imputed by scBFP

	Model input	Big-O
scBFP	X, A^{cell}, A^{gene}	$\mathcal{O}(BC) + \mathcal{O}(BG) + \mathcal{O}(\mathcal{E}_{gene}) + \mathcal{O}(\mathcal{E}_{cell})$
MAGIC	X, A^{cell}	$\mathcal{O}(GC) + \mathcal{O}(\mathcal{E}_{cell})$
ALRA	X	$\mathcal{O}(GC) + \mathcal{O}(Gk + k^2 + Ck)$
SAVER	X	$\mathcal{O}(GC) + \mathcal{O}(G) + \mathcal{O}(C)$
DCA	X, θ	$\mathcal{O}(GC) + O(\theta)$
AutoClass	X, θ	$\mathcal{O}(GC) + O(\theta)$
scGNN 2.0	X, A^{cell}, θ	$\mathcal{O}(GC) + \mathcal{O}(\mathcal{E}_{cell}) + O(\theta)$
scGCL	X, A^{cell}, θ	$\mathcal{O}(GC) + \mathcal{O}(\mathcal{E}_{cell}) + O(\theta)$

Supplementary Table S8: Memory Complexity Comparison. Let $X \in \mathbb{R}^{G \times C}$ denote the gene-cell count matrix, where G is the number of genes and C is the number of cells. Your sentence is mostly clear but can be refined for enhanced readability and mathematical precision. Let $A^{\text{cell}} \in \mathbb{R}^{C \times C}$ and $A^{\text{gene}} \in \mathbb{R}^{G \times G}$ represent the cell-cell and gene-gene adjacency matrices, respectively. The number of cell edges, denoted as $\mathcal{E}_{\text{cell}}$, and the number of gene edges, denoted as $\mathcal{E}_{\text{gene}}$, are responsible for determining the memory complexity when these matrices are stored in a sparse format. Additionally, let θ represent the trainable model parameters in each deep-learning-based method. In this context, the proposed method is equipped with a feature-wise batch training technique using a batch size B. Consequently, the memory complexity associated with matrix multiplication between the feature matrix (X) and the adjacency matrix ($A^{\cdot}$) is improved. This efficiency is notable compared to other graph-based baseline methods, which typically perform multiplication using the full size of both matrices.

References

[1] Maayan B, Adrian V, Samuel L. W, *et al.* A single-cell transcriptomic map of the human and mouse pancreas reveals inter-and intra-cell population structure. *Cell systems* **3**, 346–360 (2016).

[2] Malte D. L, Maren B, Kridsadakorn C, *et al.* Benchmarking atlas-level data integration in single-cell genomics. *Nature methods* **19**, 41–50 (2022).

[3] Allon M. K, Linas M, Ilke A, *et al.* Droplet barcoding for single-cell transcriptomics applied to embryonic stem cells. *Cell* **161**, 1187–1201 (2015).

[4] Xiaoping H, Renying W, Yincong Z, *et al.* Mapping the mouse cell atlas by microwell-seq. *Cell* **172**, 1091–1107 (2018).

[5] Amit Z, Ana B. M, Simone C, *et al.* Cell types in the mouse cortex and hippocampus revealed by single-cell rna-seq. *Science* **347**, 1138–1142 (2015).

[6] Junyue C, Jonathan S. P, Vijay R, *et al.* Comprehensive single-cell transcriptional profiling of a multicellular organism. *Science* **357**, 661–667 (2017).

[7] Evan Z. M, Anindita B, Rahul S, *et al.* Highly parallel genome-wide expression profiling of individual cells using nanoliter droplets. *Cell* **161**, 1202–1214 (2015).

[8] F Alexander W, Philipp A, Fabian J. T. Scanpy: large-scale single-cell gene expression data analysis. *Genome biology* **19**, 1–5 (2018).

[9] EA F, PJ G, MS G, *et al.* The encode (encyclopedia of dna elements) project. *Science* **306**, 636–640 (2004).

[10] Huipeng L, Elise T. C, Debarka S, *et al.* Reference component analysis of single-cell transcriptomes elucidates cellular heterogeneity in human colorectal tumors. *Nature genetics* **49**, 708–718 (2017).

[11] Saiful I, Amit Z, Simon J, *et al.* Quantitative single-cell rna-seq with unique molecular identifiers. *Nature methods* **11**, 163–166 (2014).

[12] Greg F, Andrew M, Masanao Y, *et al.* Mast: a flexible statistical framework for assessing transcriptional changes and characterizing heterogeneity in single-cell rna sequencing data. *Genome biology* **16**, 1–13 (2015).

[13] Yoav B, Yosef H. Controlling the false discovery rate: a practical and powerful approach to multiple testing. *Journal of the Royal statistical society: series B (Methodological)* **57**, 289–300 (1995).

[14] Christoph H, Rahul S. Normalization and variance stabilization of single-cell rna-seq data using regularized negative binomial regression. *Genome Biology* **20**, 296 (2019).

[15] Yuhan H, Stephanie H, Erica A-N, *et al.* Integrated analysis of multimodal single-cell data. *Cell* (2021). URL `https://doi.org/10.1016/j.cell.2021.04.048`.

[16] Michael A, Catherine A. B, Judith A. B, *et al.* Gene ontology: tool for the unification of biology. *Nature Genetics* **25**, 25–29 (2000).

[17] Suzi A. A, James B, Seth C, *et al.* The gene ontology knowledgebase in 2023. *Genetics* **224**, iyad031 (2023). URL `https://doi.org/10.1093/genetics/iyad031`.

[18] Minoru K, Yoko S, Masayuki K, *et al.* Kegg as a reference resource for gene and protein annotation. *Nucleic Acids Research* **44**, D457–D462 (2015). URL `https://doi.org/10.1093/nar/gkv1070`.

[19] Wajid J. *enrichR: Provides an R Interface to 'Enrichr'* (2023). R package version 3.2.

[20] David v. D, Roshan S, Juozas N, *et al.* Recovering gene interactions from single-cell data using data diffusion. *Cell* **174**, 716–729.e27 (2018).

[21] George C. L, Jun Z, Manolis R, *et al.* Zero-preserving imputation of single-cell rna-seq data. *Nature communications* **13**, 192 (2022).

[22] Mo H, Jingshu W, Eduardo T, *et al.* Saver: gene expression recovery for single-cell rna sequencing. *Nature methods* **15**, 539–542 (2018).

[23] Gökcen E, Lukas M. S, Maria M, *et al.* Single-cell rna-seq denoising using a deep count autoencoder. *Nature communications* **10**, 390 (2019).

[24] Hui L, Cory R. B, Weijun L. A universal deep neural network for in-depth cleaning of single-cell rna-seq data. *Nature Communications* **13**, 1901 (2022).

[25] Juexin W, Anjun M, Yuzhou C, *et al.* scgnn is a novel graph neural network framework for single-cell rna-seq analyses. *Nature communications* **12**, 1882 (2021).

[26] Zehao X, Jiawei L, Wanwan S, *et al.* scgcl: an imputation method for scrna-seq data based on graph contrastive learning. *Bioinformatics* **39**, btad098 (2023).
